# Safety evaluation of irinotecan: a real-world disproportionality analysis using FAERS and JADER databases during the time period 2004-2024

**DOI:** 10.3389/fphar.2025.1516449

**Published:** 2025-06-09

**Authors:** Siyu Lou, Huayou Chen, Zhiwei Cui, Xiyuan Zhang, Chengyu Zhu, Linmei Zhou, Yingyong Ou, Fan Zou

**Affiliations:** ^1^ Department of Respiratory and Critical Care Medicine, Affiliated Hospital of Zunyi Medical University, Zunyi, China; ^2^ The Second Affiliated Hospital of Hainan Medical University, Haikou, China; ^3^ Department of Obstetrics and Gynecology, the First Affiliated Hospital of Xi’an Jiaotong University, Xi’an, China; ^4^ Department of General Medicine, Yanan university Affiliated hospital, Yanan, China

**Keywords:** irinotecan, real-world pharmacovigilance analysis, FAERS, JADER, adverse drug event

## Abstract

**Introduction:**

Irinotecan is a widely used chemotherapeutic agent for treating colorectal, pancreatic, and ovarian cancers. Despite its therapeutic efficacy, the safety profile of irinotecan necessitates continuous pharmacovigilance due to its association with severe adverse drug events (ADEs). Given its global use, cross‐national signal detection may reveal region‐specific risks or unrecognized adverse effects.

**Methods:**

We conducted a retrospective pharmacovigilance analysis of irinotecan‐associated ADEs using two large spontaneous reporting systems: the U.S. FDA Adverse Event Reporting System (FAERS) and the Japan Adverse Drug Event Report (JADER) database. ADE reports between 2004 and 2024 were extracted. Disproportionality analyses were performed using four methods: Reporting Odds Ratio (ROR), Proportional Reporting Ratio (PRR), Bayesian confidence propagation neural network (BCPNN), and Multi‐item gamma Poisson shrinker (MGPS).

**Results:**

A total of 11,344 ADE reports from FAERS and 7,822 from JADER were identified. These reports involved 27 system organ classes (SOCs). In FAERS, the most frequently affected SOC was gastrointestinal disorders (n = 6,888), while in JADER it was blood and lymphatic system disorders (n = 3,389). Disproportionality analysis revealed 388 and 67 preferred terms (PTs) significantly associated with irinotecan in FAERS and JADER, respectively, with 38 overlapping signals. These included both expected ADEs (e.g., neutropenia, diarrhea, thrombocytopenia, stomatitis) and unexpected signals such as second primary malignancies, hyperammonaemia, and hiccups. Notable FAERS-specific signals included skin toxicity (n=100, ROR 33.89 (27.79-41.34), PRR 33.80, EBGM05 28.03, IC025 4.76), aphasia [n=65, ROR 3.57 (2.8‐4.55), PRR 3.56, EBGM05 2.90, IC025 1.47], and hepatic failure [n=56, ROR 3.09 (2.38‐4.02), PRR 3.09, EBGM05 2.48, IC025 1.24], while JADER-specific signals included fatigue [n=73, ROR 4.69 (3.71‐5.93), PRR 4.67, EBGM05 3.57, IC025 0.51], hyperammonaemia [n=67, ROR 7.24 (5.56‐9.27), PRR 7.21, EBGM05 5.32, IC025 1.10], and cholinergic syndrome [n=27, ROR 5.54 (3.76-8.16), PRR 5.53, EBGM05 3.61, IC025 0.74]. Over half of all reported ADEs occurred within one month of irinotecan administration (53.1% in FAERS, 61.7% in JADER). The median time to onset was 28 days [IQR 9‐76] in FAERS and 17 days [IQR 9‐57] in JADER.

**Discussion:**

This comparative analysis revealed multiple consistent and unexpected signals related to irinotecan use. The findings emphasize the importance of region‐specific pharmacovigilance and the need for heightened awareness of both labeled and unlabeled toxicities. Our results support continued monitoring and further investigation into temporal patterns and regional differences in irinotecan-related adverse events to enhance clinical safety.

## 1 Introduction

Irinotecan, with the molecular formula C_33_H_38_N_4_O_6_, is a chemotherapy drug derived from camptothecin, which was first approved for cancer treatment in 1994. Topoisomerase I (Topo1) is an essential enzyme for DNA replication, responsible for alleviating the topological stress generated during DNA replication and transcription. It does so by cleaving, relaxing, and re-ligating double-stranded DNA structures (Pommier, 2006). The anticancer activity of irinotecan is based on its ability to inhibit Topo1, which induces cytotoxicity by trapping the enzyme on DNA, ultimately leading to cell death (Hahn et al., 2019). While irinotecan can be used as a monotherapy, it is more commonly administered in combination with other cytotoxic agents (e.g., 5-fluorouracil and oxaliplatin) or monoclonal antibodies (e.g., cetuximab and bevacizumab). These combinations are particularly effective in treating metastatic or advanced solid tumors such as colorectal, gastric, pancreatic, and ovarian cancers (Di Desidero et al., 2017; Chen and Jiang, 2019; Hahn et al., 2019; Lu et al., 2019). In metastatic colorectal cancer (mCRC), patients who received irinotecan as part of second-line therapy following a 5-fluorouracil-based regimen demonstrated significantly longer survival compared to those treated with 5-fluorouracil and calcium folinic acid alone (Cunningham et al., 1998; Rougier et al., 1998). In pancreatic cancer, the combination of 5-fluorouracil, calcium folinic acid, irinotecan, and oxaliplatin (FOLFIRINOX) has shown superior efficacy over gemcitabine monotherapy, with a median survival of 11.1 months compared to 6.8 months (Conroy et al., 2011). Liposomal irinotecan was recently approved as a second-line therapy for metastatic pancreatic cancer in patients who have experienced disease progression after receiving gemcitabine treatment (de Man et al., 2018; Frampton, 2020).

The recommended dose of irinotecan is determined based on body surface area. For monotherapy, irinotecan doses range from 50 to 350 mg/m^2^, while in combination chemotherapy, the doses typically fall between 180 and 240 mg/m^2^ (Algeciras-Schimnich et al., 2008; Etienne-Grimaldi et al., 2015). In the FOLFIRI regimen (5-fluorouracil, leucovorin, and escalated doses of irinotecan), the recommended irinotecan dose is 180 mg/m^2^ every 2 weeks (Lu et al., 2014). After drug infusion, irinotecan blood concentrations decrease rapidly, with SN-38 levels peaking within 2 hours post-administration (Sasaki et al., 1995). The metabolism of irinotecan occurs in three major steps. Initially, the water-soluble prodrug is transformed into its active metabolite, SN-38, through the action of hepatic carboxylesterases. Following this, SN-38 is detoxified by uridine diphosphate-glucuronosyltransferase (*UGT1A1*), converting it into its inactive form, SN-38-glucuronide (SN-38G). Finally, bacterial β-glucuronidase reactivates SN-38G in the intestine, enabling the localized reformation of SN-38 before it is reabsorbed into the bloodstream (Bailly, 2019; Kciuk et al., 2020). The therapeutic effects and toxicity of irinotecan are largely attributed to SN-38, which is reported to be 100 times more cytotoxic than irinotecan itself.

Irinotecan treatment is often associated with dose-limiting toxicities, primarily diarrhea and hematological toxicities (Alimonti et al., 2004; Kweekel et al., 2008; Lam et al., 2016). According to the National Cancer Institute’s Common Toxicity Criteria, late-onset diarrhea occurs in up to 87% of patients receiving irinotecan chemotherapy, with severe grade 3 or four diarrhea observed in approximately 40% of cases (Xu et al., 2024). Other commonly reported adverse effects include fatigue and vomiting (Huisman et al., 2016). Additionally, rare toxicities such as irinotecan-induced dysarthria and retinopathy have been documented (Boilève et al., 2019a; Zhen et al., 2019). The severity of irinotecan-related toxicities depends on the specific treatment regimen, dosage, and various clinical factors, including age, body weight, sex, co-administered drugs, and pharmacogenetic variability (Algeciras-Schimnich et al., 2008; Kweekel et al., 2008). These toxicities can lead to treatment interruption or discontinuation, thereby compromising the patient’s prognosis and quality of life.

Spontaneous Reporting Systems (SRS) provide valuable safety information on the real-world use of drugs in specific populations and are a crucial source for detecting adverse reactions that may not have been identified during clinical trials(Noguchi et al., 2021). The U.S. Food and Drug Administration (FDA) Adverse Event Reporting System (FAERS) and the Japan Adverse Drug Event Report (JADER) system are two well-established SRSs, each collecting a large volume of adverse event reports primarily from U.S. and Japanese cohorts, respectively(Okada et al., 2019; van Hasselt et al., 2020). These systems enable continuous monitoring and tracking of adverse events through pharmacovigilance studies. It is crucial to acknowledge that current clinical trials may struggle to fully capture the long-term safety of irinotecan due to constraints like small sample sizes, short observation periods, and strict inclusion criteria. Therefore, there is a strong need for pharmacovigilance studies using real-world data to thoroughly evaluate irinotecan’s safety profile. To address this need, we analyzed irinotecan-associated adverse event reports from the FAERS and JADER databases to conduct a thorough assessment and comparison of the drug’s safety across two distinct populations. This pharmacovigilance study is the first to comprehensively quantify and visualize the safety profile of irinotecan using data from the FAERS and JADER databases, identify new safety signals not previously listed on the drug label, and estimate the timing of ADE occurrence. By identifying new safety signals and detailing the timing of adverse drug event (ADE) reports, this pharmacovigilance study aims to provide valuable reference information to inform the clinical use of irinotecan.

## 2 Materials and methods

### 2.1 Data source and collection

FAERS is a platform where healthcare professionals, pharmaceutical companies, patients, and other individuals can upload adverse event reports, which contributes to post-market safety monitoring of drugs (Sakaeda et al., 2013). The FAERS database consists of eight types of files: demographic and administrative information, medication details, usage indications, report sources, start and end dates of the medication, patient prognosis, reports of ineffective therapy, and adverse events. Specific data can be accessed via the FDA website (https://fis.fda.gov/extensions/FPD-QDE-FAERS/FPD-QDE-FAERS.html). The architecture of the FAERS database establishes a connection between each data file via a unique identification number.

**FIGURE 1:**
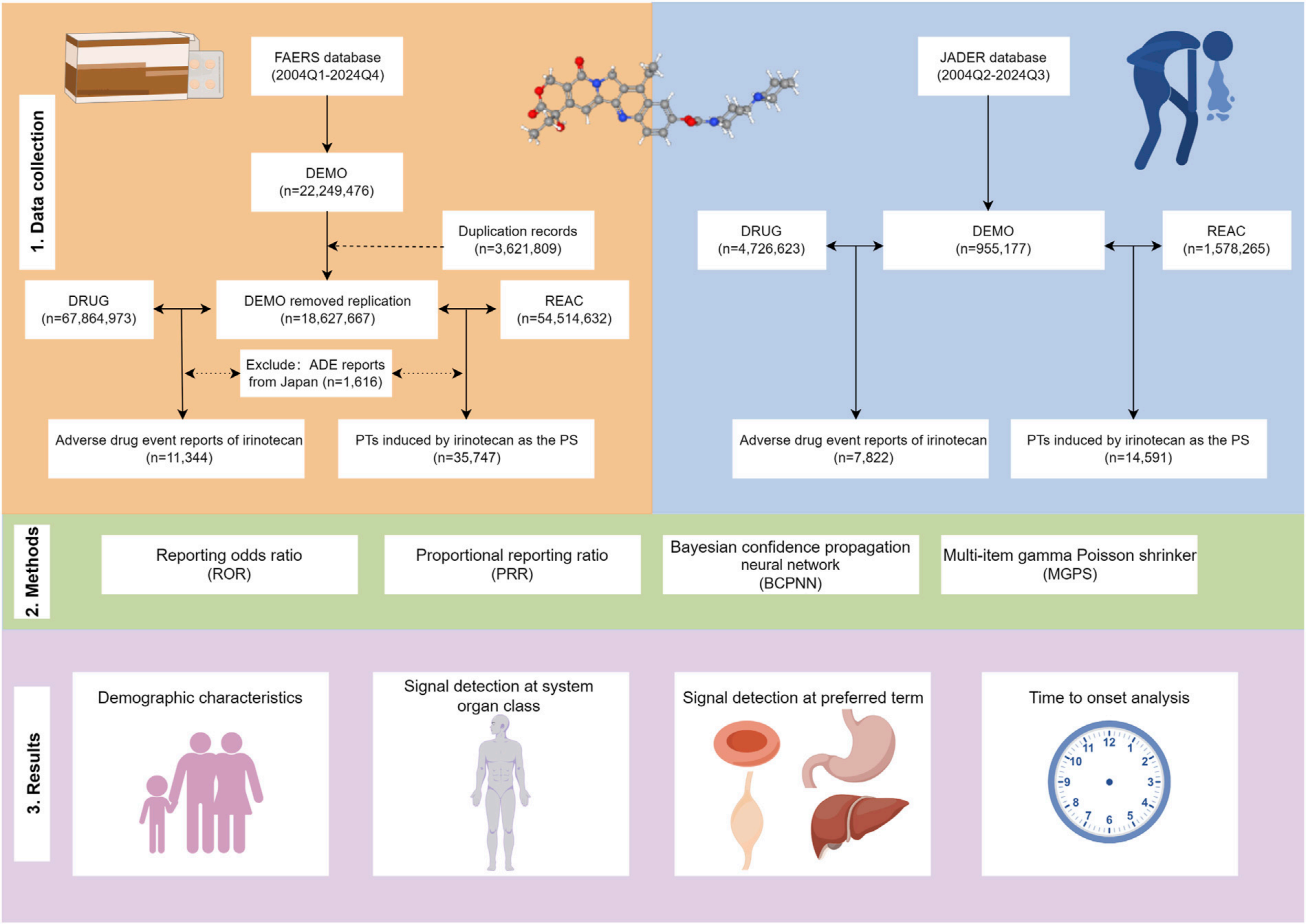
Flowchart of the entire study, including data cleaning, analysis methods, and main results. FAERS: The U.S. FDA Adverse Event Reporting System; JADER: Japanese Adverse Drug Event Report; Q1: first quarter; Q2: second quarter; Q3: third quarter; Q4: fourth quarter; PT: preferred term; PS: primary suspect.

The JADER database includes data on cases reported by pharmaceutical companies and medical institutions since the second quarter of 2004. The JADER database consists of adverse event reports voluntarily submitted by pharmaceutical companies or healthcare institutions. It is divided into four categories: DEMO, DRUG, REAC, and HIST, which contain information on patient demographics, used drugs, adverse events, and primary diseases, respectively. Data from the JADER database were obtained from the Drug and Medical Device Administration website (https://www.pmda.go.jp/index.html). Informed consent or ethical approval was not necessary for this study, as both FAERS and JADER data are publicly accessible, and the patient information in the ADE reports is anonymized. The FAERS data used in this study were collected from the first quarter of 2004 to the fourth quarter of 2024. The search terms included ‘IRINOTECAN’, ‘IRINOTECAN HCL’, ‘IRINOTECAN HYDROCHLORIDE’, ‘CPT 11 IRINOTECAN’, ‘CPT 11’, ‘CAMPTOSAR’, ‘CAMPTO’, and other generic and brand names. Similarly, in JADER, we searched for ‘イリノテカン’,'塩酸イリノテカン',和'イリノテカン塩酸塩水和物' from the second quarter of 2004 (the earliest available data in JADER) to the third quarter of 2024. The flowchart of the entire study is presented in Figure 1.

Duplicate reports exist because the FAERS database is updated quarterly. To address duplicate reports submitted by different sources, we performed deduplication as recommended by the FDA: In the DEMO file, we selected the PRIMARYIDs, CASEIDs, and FDA_DTs, subsequently sorting them by CASEIDs, FDA_DTs, and PRIMARYIDs. (1) if CASEIDs were identical, the most recent FDA_DT was selected; and (2) if both CASEIDs and FDA_DTs were the same, the higher PRIMARYID was chosen (Cui et al., 2023). Additionally, since the first quarter of 2019, each quarterly data package includes a list of deleted reports, and subsequently, we remove the reports based on the CASEIDs listed in the deleted report list (Gu et al., 2024). These rigorous approaches, in accordance with FDA guidelines, successfully removed duplicate reports and strengthened the reliability of our subsequent analyses. We extracted the ADE reports related to irinotecan from both databases and further screened these reports by retaining only those with the role code of primary suspect (PS). This process involved excluding reports related to drug-drug interactions, concomitant medications, secondary suspect medications, and other unknown medications that could potentially cause ADEs. Considering that the FAERS database includes ADE reports from Japan, we excluded the 1,616 ADE reports from Japan in FAERS to prevent duplication with those submitted to JADER. In both databases, all adverse events are coded according to the preferred term (PT) and system organ class (SOC) of the Medical Dictionary for Regulatory Activities (MedDRA) (version 27.1) (Zhu et al., 2025). The 3D structure and molecular formula of irinotecan are derived from PubChem (https://pubchem.ncbi.nlm.nih.gov/) (Kim et al., 2023).

### 2.2 Signal mining

Data mining algorithms are widely used for post-marketing safety monitoring and reassessment of drugs in SRS. In pharmacovigilance, disproportionality analysis is a powerful tool for detecting disproportionate reporting signals related to irinotecan (Caster et al., 2020). In our study, we applied both Bayesian methods (Bayesian confidence propagation neural network [BCPNN] and Multi-item gamma Poisson shrinker [MGPS]) and frequentist methods (including Reporting Odds Ratio [ROR] and Proportional Reporting Ratio [PRR]) to assess the relationship between irinotecan and ADEs(Bate et al., 1998; Evans et al., 2001; Szarfman et al., 2002; van Puijenbroek et al., 2002).Specifically, ROR is a widely used signal detection tool in pharmacovigilance, known for its simple calculation and ease of understanding, making it particularly suitable for preliminary signal detection in large databases (Rothman et al., 2004). The PRR method calculates statistical indicators by comparing the risk ratio (RR) of the target drug with the RR of the corresponding adverse event in the control group; however, small denominators can lead to significant fluctuations in results (Hai et al., 2025). The BCPNN method is based on Bayesian principles to construct a variable relationship model, combining the disproportionate reporting method with Bayesian theory, making it suitable for large-scale data analysis and capable of addressing missing data. Its advantage lies in quantifying uncertainty, thus enhancing the sensitivity and specificity of signal detection (Bate et al., 1998). MGPS detects potential signals by performing empirical Bayesian shrinkage estimation on report data, and its strength lies in handling rare events and small sample data, providing more robust signal detection results (Szarfman et al., 2002). This study combines four algorithms and performs cross-validation to fully leverage the strengths of each algorithm, validate results from multiple perspectives, minimize the risk of false positives, and improve the ability to detect potential rare adverse reactions. In our analysis, we defined a positive signal as one that meets the thresholds of all four methods simultaneously (Table 1). Unexpected signals were identified as those not listed in the drug label. To ensure methodological transparency and reproducibility, this study adhered to the READUS-PV guideline (Fusaroli et al., 2024a; b).

**TABLE 1:** The methods and thresholds for ROR, PRR, BCPNN, and EBGM are outlined. a: number of reports featuring both the specified drug and target adverse events; b: number of reports involving other adverse events alongside the specified drug; c: reports of target adverse events involving other drugs; d: reports involving other drugs and non-targeted adverse events. ROR: reporting odds ratio; PRR: proportional reporting ratio; BCPNN: Bayesian confidence propagation neural network; EBGM: empirical Bayesian geometric mean; 95% CI: 95% confidence interval; χ^2^: chi-squared; IC: information component; IC025: Information Component 2.fifth percentile; E (IC): expected IC; V(IC): variance of IC; EBGM05: Empirical Bayes Geometric Mean fifth percentile.

Drug category	Target adverse drug event	Non-target adverse drug event	Sums
Irinotecan	a	b	a+b
Non-irinotecan	c	d	c + d
Total	a+c	b + d	a+b + c + d

Methods	Formula	Threshold
ROR	$ROR=\frac{a/c}{b/d}$	a≥3
	$SE\left(lnROR\right)=\sqrt{\left(\frac{1}{a}+\frac{1}{b}+\frac{1}{c}+\frac{1}{d}\right)}$	
	$95\%\text{CI}=e^{\ln\left(\text{ROR}\right)\pm 1.96\text{se}}$	$95\%\text{CI}\left(\text{lower limit}\right)>1$
PRR	$\text{PRR}=\frac{a/\left(a+b\right)}{c/\left(c+d\right)}$	a≥3
	χ2 = [(ad-bc)^2^](a+b + c + d)/[(a+b)(c + d)(a+c)(b + d)]	χ2≥4
BCPNN	$\text{IC}=\log_{2}\frac{p\left(x,y\right)}{p\left(x\right)p\left(y\right)}=\log_{2}\frac{a\left(a+b+c+d\right)}{\left(a+b\right)\left(a+c\right)}$	IC025>0
	$E\left(\text{IC}\right)=\log_{2}\frac{\left(a+\gamma 11\right)\left(a+b+c+d+\alpha \right)\left(a+b+c+d+\beta \right)}{\left(a+b+c+d+\gamma \right)\left(a+b+\alpha 1\right)\left(a+c+\beta 1\right)}$	
	$V\left(\text{IC}\right)=\frac{1}{{\left(\ln2\right)}^{2}}\left[\frac{\left(a+b+c+d\right)-a+\gamma -\gamma 11}{\left(a+\gamma 11\right)\left(1+a+b+c+d+\gamma \right)}+\frac{\left(a+b+c+d\right)-\left(a+b\right)+a-\alpha 1}{\left(a+b+\alpha 1\right)\left(1+a+b+c+d+\alpha \right)}+\frac{\left(a+b+c+d+\alpha \right)-\left(a+c\right)+\beta -\beta 1}{\left(a+b+\beta 1\right)\left(1+a+b+c+d+\beta \right)}\right]$	
	$\gamma =\gamma 11\frac{\left(a+b+c+d+\alpha \right)\left(a+b+c+d+\beta \right)}{\left(a+b+\alpha 1\right)\left(a+c+\beta 1\right)}$	
	$\text{IC}-2\text{SD}=E\left(\text{IC}\right)-2\sqrt{V\left(\text{IC}\right)}$	
EBGM	$\text{EBGM}=\frac{a\left(a+b+c+d\right)}{\left(a+c\right)\left(a+b\right)}$	EBGM05>2
	$\text{SE}\left(\text{lnEBGM}\right)=\sqrt{\left(\frac{1}{a}+\frac{1}{b}+\frac{1}{c}+\frac{1}{d}\right)}$	
	$95\%\text{CI}=e^{\ln\left(\text{EBGM}\right)\pm 1.96\text{se}}$	

### 2.3 Classification and prioritization of relevant disproportionality signals

For all positive ADE signals, based on previously published literature(Cecco et al., 2024), we assessed the clinical priority of the signals according to the following criteria (see Table 2).

1. Clinical relevance: We referred to the “Important Medical Events (IMEs)” list (serious events, version 28.0) and the “Designated Medical Events (DMEs)” list (rare but serious events potentially induced by drugs) provided by the European Medical Agency (https://www.ema.europa.eu/en/human-regulatory-overview/research-development/pharmacovigilance-research-development/eudravigilance/eudravigilance-system-overview#reference-sources-and-services-7044, Accessed 08 April 2025).
2. Reporting Rate: The proportion of a specific ADE compared to other ADEs. We used the following traditional classification standards: very common (>10%), common (1%–10%), and uncommon (<1%), to maintain consistency with clinical trial classifications.
3. Signal Stability: Consistency/robustness of disproportionate signals across multiple algorithms. The highest score was given when a disproportionate signal appeared in at least three algorithms.
4. Reported case fatality rate: The proportion of reports of the adverse event that recorded death. Given that mortality is typically higher in cancer treatments, distinguishing between drug-induced adverse events and those resulting from natural disease progression can be challenging. In this study, the highest score was assigned only when the fatality rate exceeded 50%.

**TABLE 2:** The criteria and relevant scores for prioritizing adverse drug events (ADEs) identified through disproportionality analysis.

Criterium	2 points	1 point	0 point
Reporting rate (cases/non-cases)	>10%	1%–10%	0%–1%
Signal stability (consistency across disproportionality analyses)	3/4 of 4	2 of 3	1 of 3
Reported case fatality rate (proportion of reports with death as outcome)	>50%	25%–50%	<25%
Clinical relevance (serious likely drug-attributable ADEs)	DME	IME	None

ADEs, adverse drug events; DME, designated medical event; IME, important medical event.

Adverse events with scores of 0–2, 3-5, and 6-8 were classified as low, medium, and high clinical priority, respectively.

### 2.4 Time to onset analysis

We defined the time to onset (TTO) of irinotecan-related ADEs as the time interval between the date of ADE occurrence (EVENT_DT) the start date of drug treatment (START_DT). In FAERS, EVENT_DT is located in the ‘DEMO’ file, while START_DT is found in the ‘THER’ file. In JADER, both EVENT_DT and START_DT are included in the 'DRUG' file. Cases with any missing dates (whether the drug start date or ADE occurrence date), inaccurate dates (where the specific day, month, or year is not clearly specified), and cases where the ADE onset date occurs before the drug treatment date will be excluded, thereby enhancing the accuracy of the analysis (Shu et al., 2023). The overall characteristics of TTOs were comprehensively evaluated using the median, quartiles, and a Weibull distribution test(Kinoshita et al., 2020). The Weibull distribution test characterizes the pattern of adverse event risk over time through the scale parameter (α) and shape parameter (β) (Mazhar et al., 2021).

## 3 Results

### 3.1 Description of baseline information of ADE report

**FIGURE 2:**
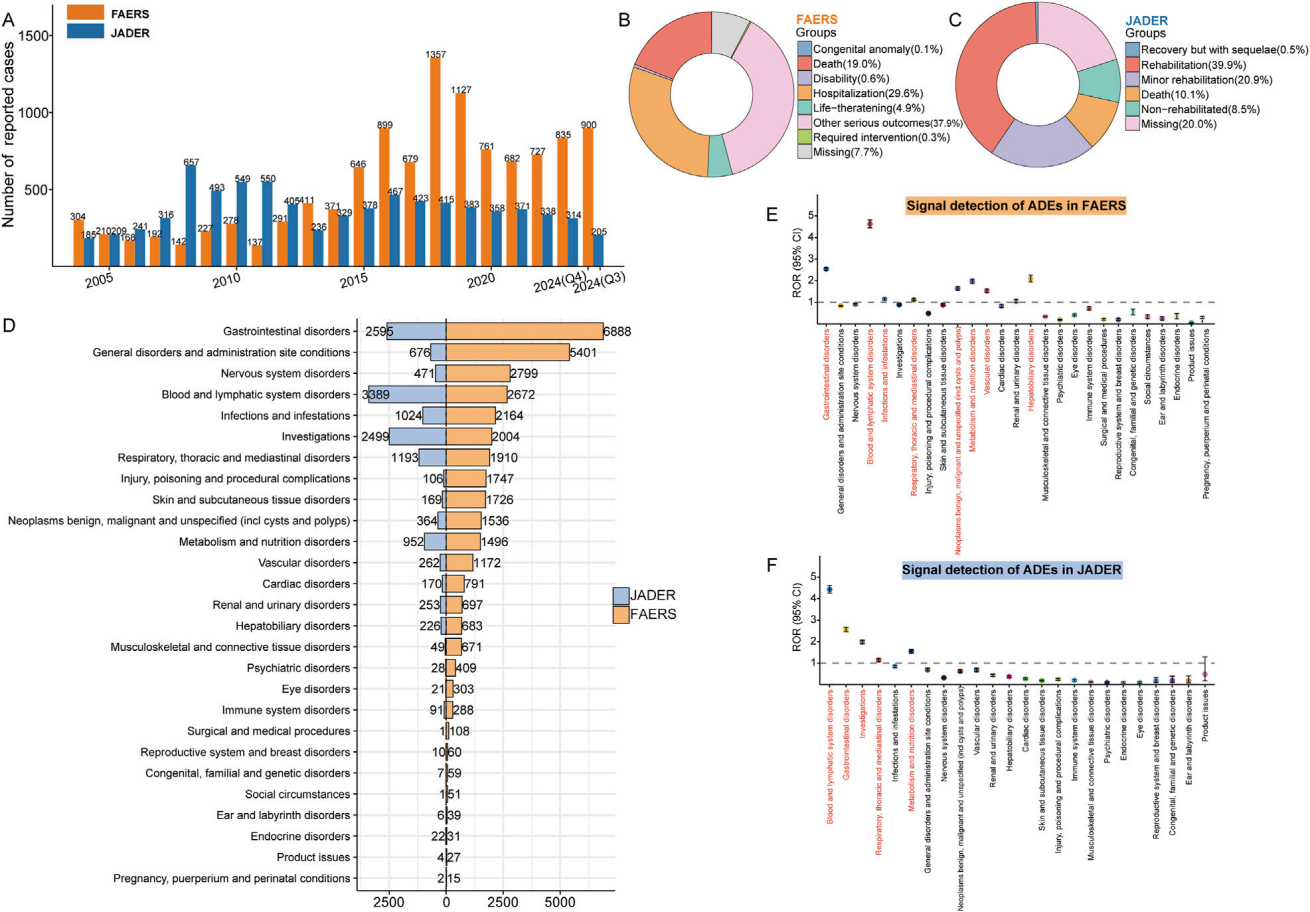
Signal detection at the SOC level. **(A)** Annual distribution of ADE reports in the FAERS and JADER databases, displayed as a bar chart. Submitter outcome data from FAERS **(B)** and JADER **(C)** were illustrated using donut plots to visually represent the distribution of reported clinical outcomes. **(D)** Number of ADE reports in FAERS and JADER at the SOC level. **(E, F)** Signal detection at the SOC level in FAERS and JADER, with ROR values and their 95% confidence intervals (95% CI) visualized. SOCs meeting the signal threshold are highlighted in red. SOC: system organ class; ADE: adverse drug event; FAERS: The U.S. FDA Adverse Event Reporting System; JADER: Japanese Adverse Drug Event Report; ROR: reporting odds ratio; Q3: third quarter; Q4: fourth quarter.

From 2004 to 2024, 11,344 and 7,822 ADE reports were submitted to the FAERS and JADER databases, respectively (Figure 1). In terms of the annual distribution of ADEs, the number of reports in the FAERS database remained consistently high after 2015, exceeding 600 reports per year. In contrast, the number of ADE reports in the JADER database remained relatively stable over the years. Notably, the number of ADEs in JADER exceeded that in FAERS from 2006 to 2012, but this trend reversed after 2013 (Figure 2A).

A demographic analysis of FAERS revealed that the percentage of ADE reports submitted by males and females was relatively balanced, with 45.6% from males and 33.7% from females. Regarding age distribution, the majority of reports (67.2%) came from individuals over 18 years old. Additionally, 34.7% of reports contained specific weight information, with the majority of submitters (29.2%) weighing between 50 and 100 kg. The top five reporting countries were the United States (27.1%), France (12.1%), Italy (9.8%), the United Kingdom (6.4%), and Canada (6.3%). Most of the reports (n = 10,114, 89.2%) were submitted by healthcare professionals, ensuring the reliability of the data. In terms of outcomes, other serious outcomes (37.9%), hospitalizations (29.6%), and deaths (19.0%) accounted for the largest proportion of reported events in FAERS (Figure 2B). The top five reported indications were metastatic colorectal cancer (16.2%), pancreatic carcinoma (10.0%), colon cancer (7.4%), colorectal cancer (6.4%), and metastatic colon cancer (3.6%) (Table 3).

**TABLE 3:** Demographic characteristics of ADEs reported in the FAERS database with irinotecan as the primary suspect drug. FAERS: The U.S. FDA Adverse Event Reporting System.

Characteristics	Case number	Case proportion, %
Sex, n (%)		
Female	3821	33.7%
Male	5176	45.6%
Unknown	1,347	20.7%
Age		
18–65 years	4307	38.0%
>65 years	3312	29.2%
Unknown	3291	29.0%
Weight		
50–100 kg	3311	29.2%
>100 kg	254	2.2%
Unknown	7410	65.3%
Reported Countries (top five)		
France	1371	12.1%
Italy	1109	9.8%
United Kingdom	724	6.4%
Canada	713	6.3%
Reported person		
Consumer	674	5.9%
Unknown	556	4.9%
Outcome		
Hospitalization	3359	29.6%
Life-threatening	552	4.9%
Disability	65	0.6%
Required intervention	32	0.3%
Death	2154	19.0%
Other serious outcomes	4304	37.9%
Congenital anomaly	5	0.0%
Unknown	873	7.7%
Indication (top five)		
Colorectal cancer metastatic	1838	16.2%
Pancreatic carcinoma	1150	10.1%
Colon cancer	843	7.4%
Colorectal cancer	730	6.4%
Colon cancer metastatic	410	3.6%

In the JADER database, ADE reports submitted by males (60.4%) were more frequent than those from females (36.4%). The majority of reports (96.4%) contained age information, with 59.0% of submitters falling within the 20–70 years age range. Similar to FAERS, the highest proportion of submitters in JADER (41.3%) weighed between 50 and 100 kg. More than 60% of the submitters experienced recovery, while 10.1% resulted in death, and 8.5% in non-recovery (Figure 2C). The top five reported indications were colon cancer (28.5%), rectal cancer (14.9%), pancreatic cancer (11.7%), colorectal cancer (10.5%), and gastric cancer (6.4%) (Table 4).

**TABLE 4:** Demographic characteristics of ADEs reported in the JADER database with irinotecan as the primary suspect drug. JADER: Japanese Adverse Drug Event Report.

Characteristics	Case number	Case proportion, %
Sex, n (%)		
Female	2,845	36.4%
Male	4,724	60.4%
Unknown	253	3.2%
Age		
20–70 years	4,615	59.0%
>70 years	2,810	35.9%
Unknown	285	3.6%
Weight		
50–100 kg	3,229	41.3%
>100 kg	4	0.1%
Unknown	3,230	41.3%
Outcome		
Recovery but with sequelae	80	0.5%
Rehabilitation	5,829	39.9%
Minor rehabilitation	3,050	20.9%
Death	1,477	10.1%
Non-rehabilitated	1235	8.5%
Missing	2920	20.0%
Indication (top five)		
Colon cancer	2,229	28.5%
Rectal cancer	1,163	14.9%
Pancreatic cancer	915	11.7%
Colorectal cancer	821	10.5%
Gastric cancer	502	6.4%

### 3.2 Signal detection at the SOC level

According to the SOC, we counted and compared ADE reports from the FAERS and JADER databases. In both cohorts, ADEs associated with irinotecan dosing spanned across 27 SOCs. The total number of PTs induced by irinotecan was 35,747 in FAERS and 14,591 in JADER. The top three SOCs with the highest number of reported cases in FAERS were “gastrointestinal disorders” (n = 6,888), “general disorders and administration site conditions” (n = 5,401), and “nervous system disorders” (n = 2,799). In contrast, the top three SOCs in JADER were “blood and lymphatic system disorders” (n = 3,389), “gastrointestinal disorders” (n = 2,595), and “investigations” (n = 2,499) (Figure 2D). A comparison of the composition ratios revealed significant differences between the two cohorts in terms of SOC distribution (chi-square, *P* < 0.0001, Supplementary Figure S1). In FAERS, certain SOCs had a significantly higher composition ratio compared to JADER, including “general disorders and administration site conditions” (15.1% vs 4.6%), “nervous system disorders” (7.8% vs 3.2%), “injury, poisoning and procedural complications” (4.9% vs 0.7%), and “skin and subcutaneous tissue disorders” (4.8% vs 1.2%). Conversely, JADER showed a higher composition ratio for SOCs such as “blood and lymphatic system disorders” (23.2% vs 7.5%), and “investigations” (17.1% vs. 5.6%).

Regarding the intensity of signal values, eight SOCs showed positive signal values in FAERS, including “gastrointestinal disorders” (ROR 2.54, 95% CI: 2.47–2.60), “blood and lymphatic system disorders” (ROR 4.63, 95% CI:4.45–4.81), “infections and infestations” (ROR 1.15, 95% CI:1.10–1.20), “respiratory, thoracic, and mediastinal disorders” (ROR 1.12, 95% CI: 1.07–1.17), “neoplasms benign, malignant, and unspecified (including cysts and polyps)” (ROR 1.64, 95% CI: 1.56–1.73), “metabolism and nutrition disorders” (ROR 1.98, 95% CI: 1.88–2.08), “vascular disorders” (ROR 1.53, 95% CI: 1.44–1.62), and “hepatobiliary disorders” (ROR 2.09, 95% CI: 1.94–2.26) (Figure 2E). In JADER, five SOCs showed positive signal values, including “blood and lymphatic system disorders” (ROR 4.43, 95% CI: 4.26–4.61), “gastrointestinal disorders” (ROR 2.57, 95% CI: 2.46–2.68), “investigations” (ROR 1.98, 95% CI: 1.90–2.07), “respiratory, thoracic, and mediastinal disorders” (ROR 1.14, 95% CI: 1.07–1.21), and “metabolism and nutrition disorders” (ROR 1.55, 95% CI: 1.45–1.65) (Figure 2F). Importantly, SOCs that met the threshold for disproportionality analysis in both cohorts included “blood and lymphatic system disorders,” “gastrointestinal disorders,” “respiratory, thoracic, and mediastinal disorders,” and “metabolism and nutrition disorders.” A detailed breakdown of SOC-level signal detection results is provided in Table 5.

**TABLE 5:** Signal detection at the SOC level in FAERS and JADER databases. Numbers one and 2 marked in the lower right corner refer to calculations results related to FAERS and JADER, respectively. FAERS: The U.S. FDA Adverse Event Reporting System; ROR: reporting odds ratio; CI: confidence interval; PRR: proportional reporting ratio; χ^2^: chi-squared; IC: information component; IC025: Information Component 2.fifth percentile; EBGM: empirical Bayes geometric mean; EBGM05: Empirical Bayes Geometric Mean fifth percentile; SOC: system organ class.

SOC name	Case number_1_	Case number_2_	ROR (95% CI)_1_	ROR (95% CI)_2_	PRR_1_	PRR_2_	EBGM (EBGM05)_1_	EBGM (EBGM05)_2_	IC (IC025)_1_	IC (IC025)_2_
Gastrointestinal disorders	6888	2595	2.54 (2.47–2.6)	2.57 (2.46–2.68)	2.24 (5163.75)	2.29 (2007.05)	2.24 (2.19)	2.26 (2.17)	1.16 (1.12)	1.18 (−0.49)
General disorders and administration site conditions	5401	676	0.84 (0.81–0.86)	0.69 (0.64–0.75)	0.86 (147.63)	0.7 (88.94)	0.86 (0.84)	0.71 (0.65)	−0.22 (−0.26)	−0.5 (−2.17)
Nervous system disorders	2799	471	0.91 (0.88–0.95)	0.32 (0.29–0.35)	0.92 (22.15)	0.34 (656.5)	0.92 (0.89)	0.34 (0.31)	−0.12 (−0.18)	−1.54 (−3.21)
Blood and lymphatic system disorders	2672	3389	4.63 (4.45–4.81)	4.43 (4.26–4.61)	4.35 (7006.03)	3.63 (6698.31)	4.35 (4.2)	3.55 (3.41)	2.12 (2.06)	1.83 (0.16)
Infections and infestations	2164	1024	1.15 (1.1–1.2)	0.85 (0.8–0.91)	1.14 (37.62)	0.86 (23.64)	1.14 (1.1)	0.87 (0.81)	0.18 (0.12)	−0.21 (−1.88)
Investigations	2004	2499	0.89 (0.85–0.93)	1.98 (1.9–2.07)	0.9 (24.37)	1.82 (996.04)	0.9 (0.87)	1.8 (1.73)	−0.15 (−0.22)	0.85 (−0.82)
Respiratory, thoracic and mediastinal disorders	1910	1193	1.12 (1.07–1.17)	1.14 (1.07–1.21)	1.11 (21.97)	1.13 (18.56)	1.11 (1.07)	1.13 (1.06)	0.15 (0.08)	0.17 (−1.49)
Injury, poisoning and procedural complications	1747	106	0.49 (0.47–0.51)	0.24 (0.2–0.29)	0.51 (888.94)	0.25 (248.78)	0.51 (0.49)	0.25 (0.21)	−0.96 (−1.03)	−2 (−3.67)
Skin and subcutaneous tissue disorders	1726	169	0.88 (0.84–0.93)	0.18 (0.15–0.21)	0.89 (25.43)	0.19 (627.7)	0.89 (0.85)	0.19 (0.16)	−0.17 (−0.24)	−2.4 (−4.06)
Neoplasms benign, malignant and unspecified (incl cysts and polyps)	1536	364	1.64 (1.56–1.73)	0.62 (0.56–0.69)	1.62 (370.91)	0.63 (81.36)	1.62 (1.55)	0.63 (0.57)	0.69 (0.62)	−0.66 (−2.33)
Metabolism and nutrition disorders	1496	952	1.98 (1.88–2.08)	1.55 (1.45–1.65)	1.94 (690.23)	1.51 (169.21)	1.93 (1.85)	1.5 (1.41)	0.95 (0.88)	0.59 (−1.08)
Vascular disorders	1172	262	1.53 (1.44–1.62)	0.68 (0.6–0.77)	1.51 (206.11)	0.68 (38.88)	1.51 (1.44)	0.69 (0.61)	0.59 (0.51)	−0.54 (−2.21)
Cardiac disorders	791	170	0.83 (0.77–0.89)	0.27 (0.23–0.32)	0.83 (27.69)	0.28 (325.73)	0.83 (0.78)	0.28 (0.24)	−0.27 (−0.37)	−1.82 (−3.49)
Renal and urinary disorders	697	253	1.05 (0.98–1.14)	0.43 (0.38–0.49)	1.05 (1.88)	0.44 (187.52)	1.05 (0.99)	0.44 (0.39)	0.07 (−0.04)	−1.18 (−2.84)
Hepatobiliary disorders	683	226	2.09 (1.94–2.26)	0.37 (0.33–0.42)	2.07 (382.82)	0.38 (236.51)	2.07 (1.95)	0.38 (0.34)	1.05 (0.94)	−1.39 (−3.05)
Musculoskeletal and connective tissue disorders	671	49	0.34 (0.32–0.37)	0.12 (0.09–0.16)	0.35 (841.09)	0.12 (311.06)	0.35 (0.33)	0.13 (0.09)	−1.5 (−1.61)	−3 (−4.67)
Psychiatric disorders	409	28	0.19 (0.17–0.21)	0.09 (0.06–0.13)	0.2 (1389.02)	0.09 (252.53)	0.2 (0.18)	0.09 (0.06)	−2.32 (−2.47)	−3.41 (−5.08)
Eye disorders	303	21	0.41 (0.37–0.46)	0.09 (0.06–0.14)	0.42 (249.55)	0.09 (183.95)	0.42 (0.38)	0.1 (0.06)	−1.26 (−1.42)	−3.39 (−5.05)
Immune system disorders	288	91	0.72 (0.64–0.8)	0.2 (0.16–0.24)	0.72 (32.39)	0.2 (294.85)	0.72 (0.65)	0.2 (0.17)	−0.48 (−0.65)	−2.3 (−3.96)
Surgical and medical procedures	108	1	0.22 (0.18–0.26)	0.02 (0–0.12)	0.22 (301.97)	0.02 (59.5)	0.22 (0.19)	0.02 (0)	−2.18 (−2.46)	−5.92 (−7.59)
Reproductive system and breast disorders	60	10	0.2 (0.16–0.26)	0.18 (0.09–0.33)	0.2 (188.86)	0.18 (38.74)	0.2 (0.16)	0.18 (0.1)	−2.3 (−2.67)	−2.5 (−4.16)
Congenital, familial and genetic disorders	59	7	0.54 (0.42–0.69)	0.19 (0.09–0.39)	0.54 (23.55)	0.19 (25.01)	0.54 (0.43)	0.19 (0.09)	−0.9 (−1.27)	−2.42 (−4.08)
Social circumstances	51	1	0.32 (0.25–0.43)	0.13 (0.02–0.94)	0.33 (71.69)	0.13 (5.71)	0.33 (0.26)	0.13 (0.02)	−1.62 (−2.02)	−2.91 (−4.58)
Ear and labyrinth disorders	39	6	0.25 (0.18–0.34)	0.18 (0.08–0.41)	0.25 (87.27)	0.18 (21.85)	0.25 (0.19)	0.18 (0.08)	−1.99 (−2.45)	−2.44 (−4.1)
Endocrine disorders	31	22	0.34 (0.24–0.48)	0.09 (0.06–0.13)	0.34 (40.5)	0.09 (207.78)	0.34 (0.25)	0.09 (0.06)	−1.57 (−2.08)	−3.47 (−5.14)
Product issues	27	4	0.05 (0.03–0.07)	0.48 (0.18–1.29)	0.05 (525.84)	0.48 (2.22)	0.05 (0.03)	0.48 (0.18)	−4.4 (−4.95)	−1.05 (−2.72)
Pregnancy, puerperium and perinatal conditions	15	2	0.1 (0.06–0.16)	0.03 (0.01–0.14)	0.1 (126.27)	0.03 (53.87)	0.1 (0.06)	0.04 (0.01)	−3.36 (−4.08)	−4.84 (−6.5)

### 3.3 Signal detection at the PT level

**FIGURE 3:**
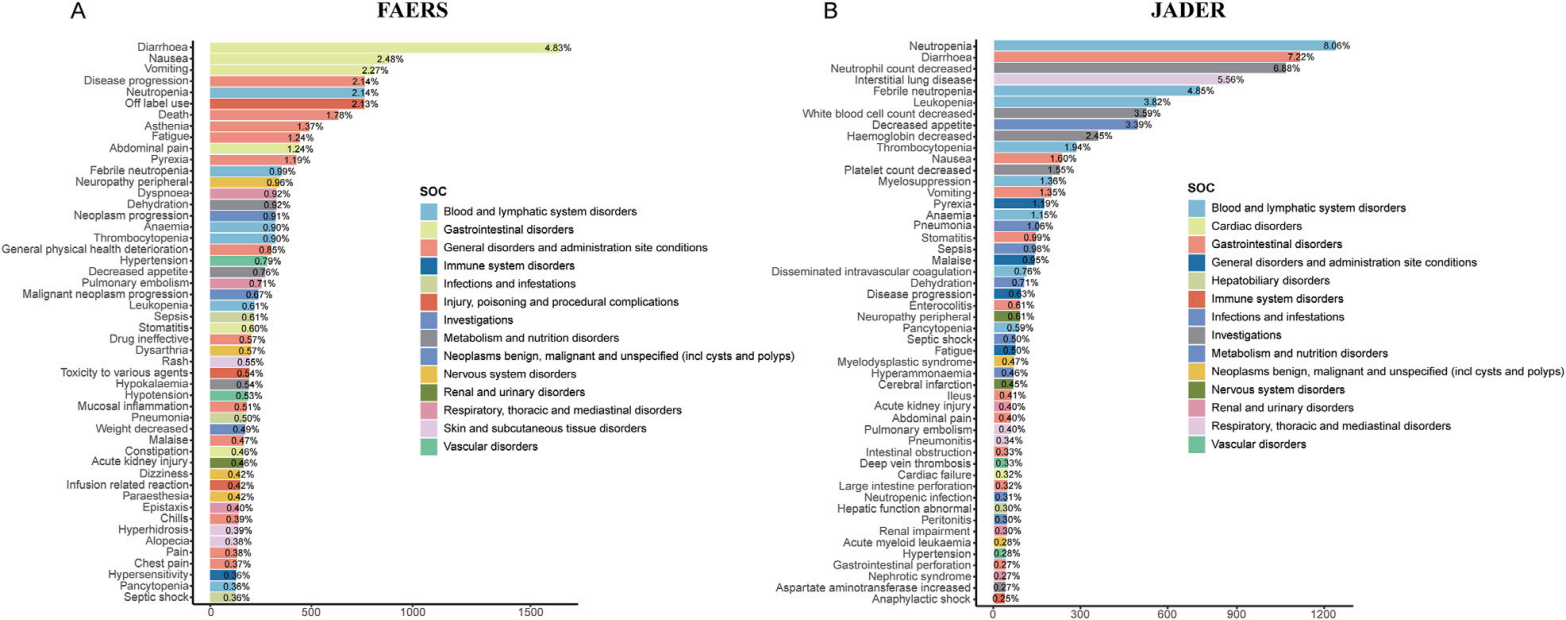
Bar plots displaying the top 50 PTs in terms of reported cases in FAERS **(A)** and JADER **(B)** databases. The color indicates the SOC of the corresponding PT. The percentage values labeled in the figure represent the proportion of cases with such ADEs out of the total reported ADEs. SOC: system organ class; PT: preferred term.

Subsequently, we performed signal detection at the PT level. By focusing solely on the number of reported cases, without considering signal intensity, we identified and listed the top 50 PT entries with the highest percentages in both cohorts. In FAERS, the top five PT signals by percentage were diarrhea (n = 1,728, 4.83%), nausea (n = 888, 2.48%), vomiting (n = 813, 2.27%), disease progression (n = 765, 2.14%), and neutropenia (n = 764, 2.14%) (Figure 3A). In JADER, the top five were neutropenia (n = 1,176, 8.06%), diarrhea (n = 1,053, 7.22%), decreased neutrophil count (n = 1,004, 6.88%), interstitial lung disease (n = 811, 5.56%), and febrile neutropenia (n = 708, 4.85%) (Figure 3B).

**FIGURE 4:**
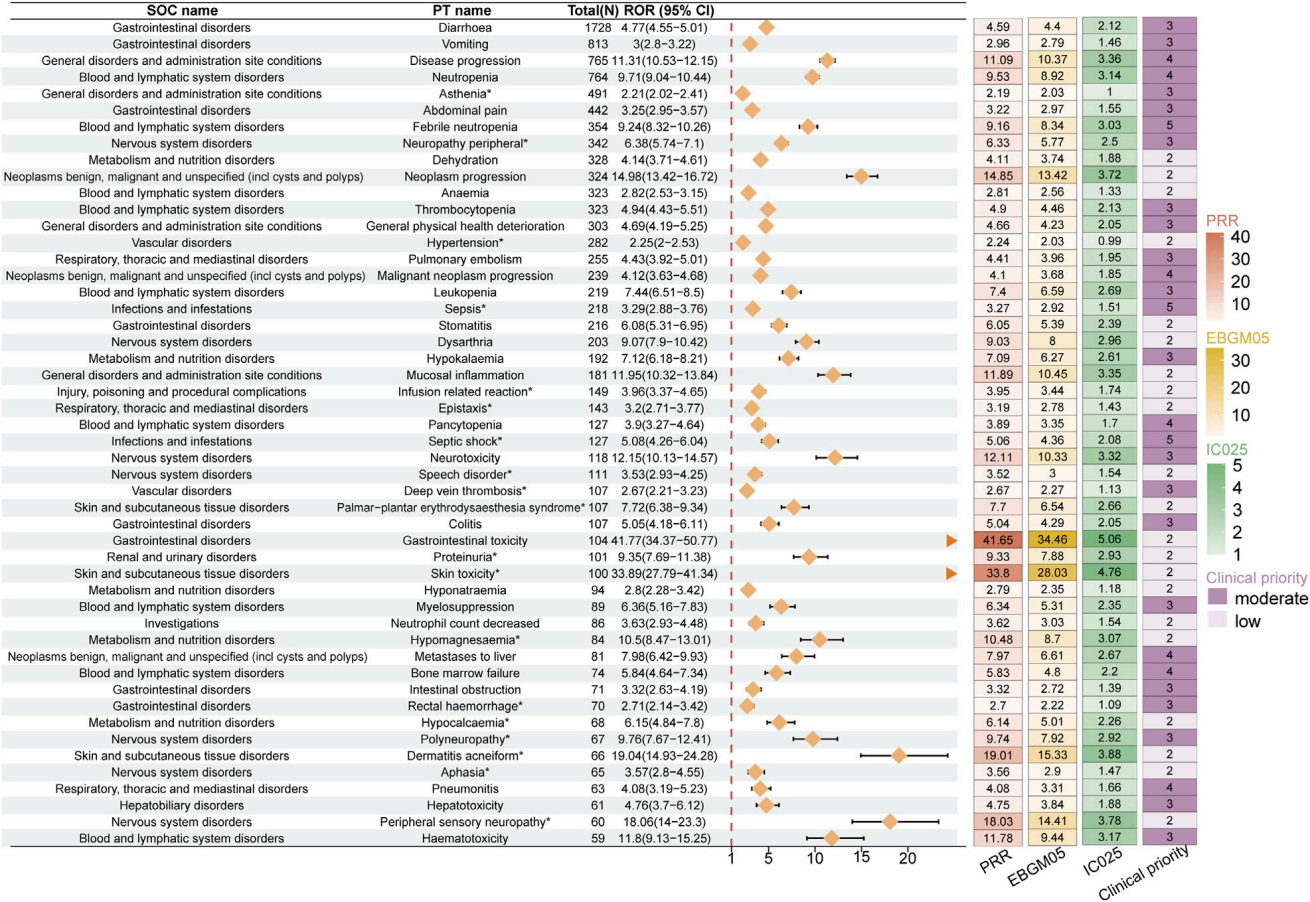
Forest plot showing the top 50 PT entries (ranked by reported cases) that simultaneously satisfy the four disproportionality methods with positive signal strength in the FAERS database. The organ arrows indicate that the lower limit of the 95% confidence interval for the ROR exceeds 20. The heatmap on the right displays the signal values under different algorithms. The darker the color, the stronger the signal value. Asterisks (*) indicate unexpected signals not listed in the drug label. PT: preferred term; FAERS: The U.S. FDA Adverse Event Reporting System.

Using the disproportionality analysis method, we identified a total of 388 signals in the FAERS database that simultaneously satisfied the positive thresholds of all four algorithms (Supplementary Table S1). In comparison, 67 signals were identified in the JADER database (Supplementary Table S2). The top five signals in FAERS, ranked by the number of cases, were diarrhea (n = 1,728, ROR 4.77, PRR 4.59, EBGM05 4.40, IC025 2.12), vomiting (n = 813, ROR 3.00, PRR 2.96, EBGM05 2.79, IC025 1.46), disease progression (n = 765, ROR 11.31, PRR 11.09, EBGM05 10.37, IC025 3.36), neutropenia (n = 764, ROR 9.71, PRR 9.53, EBGM05 8.92, IC025 3.14), and asthenia (n = 491, ROR 2.21, PRR 2.19, EBGM05 2.03, IC025 1.00) (Figure 4). Additionally, we identified several unexpected signals not previously mentioned in the drug label, such as peripheral neuropathy (n = 342, ROR 6.38, PRR 6.33, EBGM05 5.77, IC025 2.50), hypertension (n = 282, ROR 2.25, PRR 2.24, EBGM05 2.03, IC025 0.99), epistaxis (n = 143, ROR 3.20, PRR 3.19, EBGM05 2.78, IC025 1.43), septic shock (n = 127, ROR 5.08, PRR 5.06, EBGM05 4.36, IC025 2.08), proteinuria (n = 101, ROR 9.35, PRR 9.33, EBGM05 7.88, IC0252.93), skin toxicity (n = 100, ROR 33.89, PRR 33.80, EBGM05 28.03, IC025 4.76), palmar-plantar erythrodysaesthesia syndrome (n = 107, ROR 7.72, PRR 7.70, EBGM05 6.54, IC025 2.66), and hypomagnesaemia (n = 84, ROR 10.5, PRR 10.48, EBGM05 8.7, IC025 3.07). In the clinical priority assessment, we identified one high clinical priority signal: hepatic failure (total score: 6), which is also an unexpected signal. Additionally, there are 226 moderate clinical priority signals, including febrile neutropenia (total score: 5), sepsis (total score: 5), and others. Furthermore, there are 161 low clinical priority signals, including dehydration (total score: 2), anaemia (total score: 2), and others (Supplementary Table S1).

**FIGURE 5:**
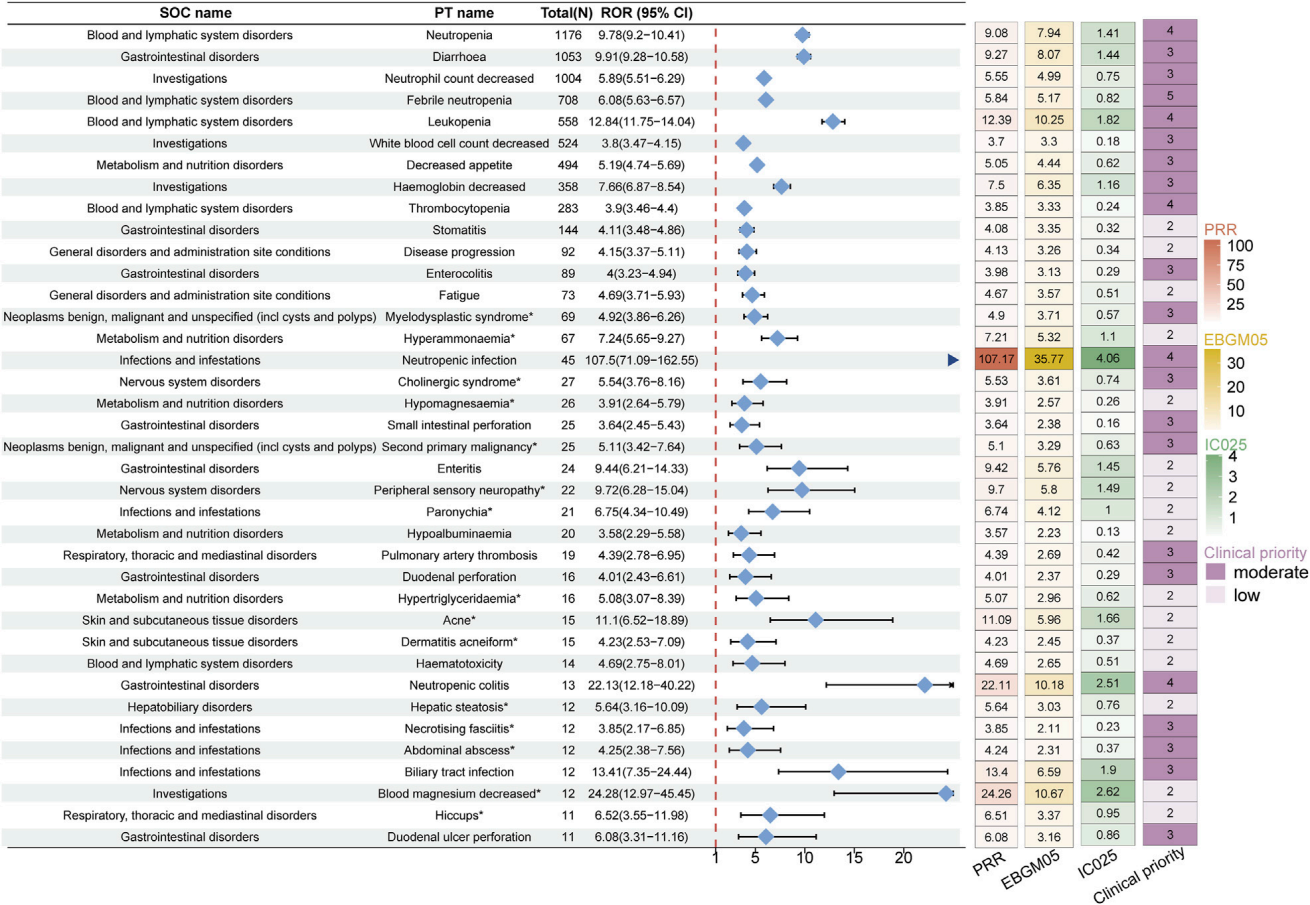
Forest plot showing PT entries that simultaneously satisfy the four disproportionality methods with positive signal strength and at least 10 reported cases in the JADER database. The blue arrow indicates that the lower limit of the 95% confidence interval for the ROR exceeds 20. The heatmap on the right displays the signal values under different algorithms. The darker the color, the stronger the signal value. Asterisks (*) indicate unexpected signals not listed in the drug label. PT: preferred term; JADER: Japanese Adverse Drug Event Report.

In the JADER database, the top five signals by number of cases were neutropenia (n = 1,176, ROR 9.78, PRR 9.08, EBGM05 7.94, IC025 1.14), diarrhea (n = 1,053, ROR 9.91, PRR 9.27, EBGM05 8.07, IC025 1.44), decreased neutrophil count (n = 1,004, ROR 5.89, PRR 5.55, EBGM05 4.99, IC025 0.75), febrile neutropenia (n = 708, ROR 6.08, PRR 5.84, EBGM05 5.17, IC025 0.82), and leukopenia (n = 558, ROR 12.84, PRR 12.39, EBGM05 10.25, IC025 1.82) (Figure 5). Unexpected signals identified in JADER included hyperammonaemia (n = 67, ROR7.24, PRR 7.21, EBGM05 5.32, IC025 1.10), myelodysplastic syndrome (n = 69, ROR 4.92, PRR 4.90, EBGM05 3.71, IC025 0.57), cholinergic syndrome (n = 27, ROR 5.54, PRR 5.53, EBGM05 3.61, IC025 0.74), hypomagnesaemia (n = 26, ROR 3.91, PRR 3.91, EBGM05 2.57, IC025 0.26), and second primary malignancy (n = 25, ROR 5.11, PRR 5.10, EBGM05 3.29, IC025 0.63). In the clinical priority assessment, we identified no high clinical priority signal. There are 39 moderate clinical priority signals, including febrile neutropenia (total score: 5), metastases to meninges (total score: 5), and others. Moreover, there are 28 low clinical priority signals, including stomatitis (total score: 2), fatigue (total score: 2), and others (Supplementary Table S2).

Notably, there was an overlap of 38 positive signals between the two databases, including 16 unexpected signals such as hypomagnesaemia, peripheral sensory neuropathy, hiccups, paronychia, second primary malignancy, and dermatitis acneiform (Supplementary Figure S2).

### 3.4 Sensitivity analysis

In clinical practice, irinotecan is frequently used in combination with platinum-based agents and fluorouracil, among others (Xu et al., 2018; Conroy et al., 2021). To exclude potential confounding effects of concomitant medications, we collected 1,577 ADE reports from FAERS after excluding all reports involving concomitant use with irinotecan. Following disproportionality analysis, persistent positive signals included diarrhea, vomiting, speech disorder, dysarthria, neutropenia, dehydration, febrile neutropenia, aphasia, gastrointestinal toxicity, increased blood bilirubin, and hepatic failure (Supplementary Table S3).

### 3.5 Time to onset analysis

**FIGURE 6:**
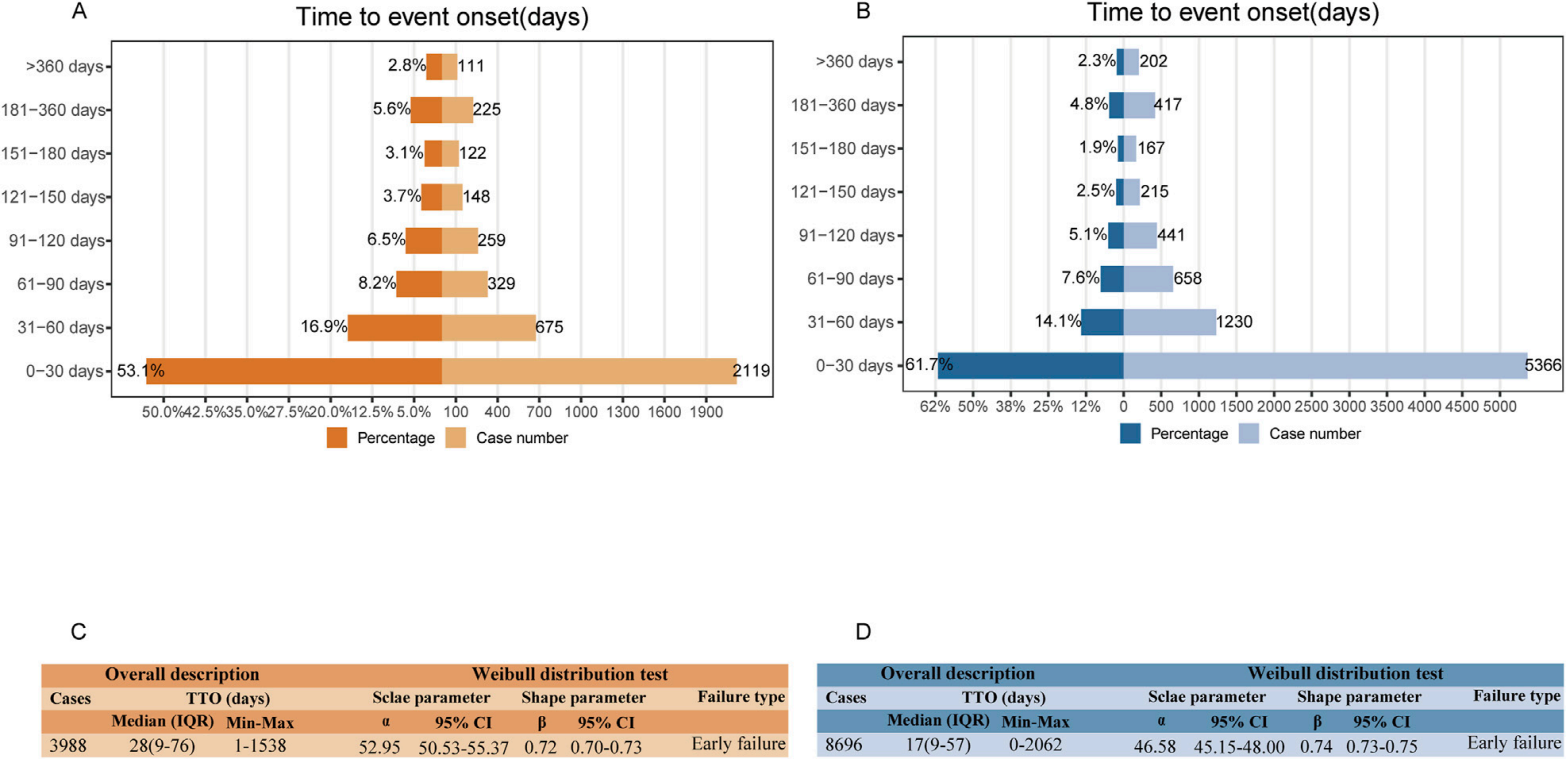
Time to onset (TTO) analysis (counted in days) of irinotecan-related ADEs. Bar graphs depict the number and proportion of ADE reports at different time intervals in FAERS **(A)** and JADER **(B)**. Overall description and Weibull distribution test analysis of valid TTO reports in FAERS **(C)** and JADER **(D)**. ADE: adverse drug event; IQR: interquartile range.

For clinicians, timely and accurate assessment and appropriate management of adverse reactions can significantly improve patients’ quality of life and functional outcomes, helping to avoid serious and potentially fatal consequences(Szebeni et al., 2018; Locke et al., 2020). To further explore this, we assessed the TTO of ADEs associated with irinotecan in both cohorts. In FAERS, we collected 3,988 reports with accurate TTO information. The median TTO was 28 days, with an interquartile range (IQR) of 9–76 days (Figure 6C). The distribution of TTOs showed that the majority of ADEs occurred within the first month of treatment (n = 2,119, 53.1%), with a decreased incidence in the second (n = 675, 16.9%) and third (n = 329, 8.2%) months. Notably, approximately 2.8% of ADEs (n = 111) were reported more than 360 days after irinotecan treatment initiation (Figure 6A). In JADER, 8,696 TTO reports were obtained, with a median TTO of 17 days (IQR: 9-57) (Figure 6D). Similar to the FAERS results, ADEs were primarily clustered within the first month of treatment (n = 5,366, 61.7%), with a declining trend over time. The percentage of ADEs reported over 360 days was 2.3% (Figure 6B).

The Weibull shape parameter test for TTO indicated that the upper limit of the 95% confidence interval (CI) for the shape parameter (β) was less than one in both FAERS (0.73) and JADER (0.75), consistent with an early failure type. This suggests that the probability of an ADE decreases gradually over time in both cohorts (Figures 6C,D).

## 4 Discussion

### 4.1 Baseline information

In 2020, colorectal cancer (CRC) resulted in over 1.9 million new cases and 900,000 deaths, ranking as the third most common cancer and the second leading cause of cancer-related deaths worldwide (Roshandel et al., 2024). The United States and China reported the highest prevalence rates, followed by Japan, Russia, India, Germany, Brazil, the United Kingdom, Italy, and France. This geographic distribution is generally consistent with the data from reporting countries in FAERS (Eng et al., 2024). The estimated median age of onset for new CRC cases is 67 years, though approximately 10% of submitters are younger than 50 (Eng et al., 2024). Most new CRC diagnoses (56.6%) and related deaths (46.6%) occur in adults aged over 70 years. However, since the early 1990s, both incidence and mortality rates have been rising among younger individuals (<50 years) across most regions of the world (Murphy and Zaki, 2024). In North America, incidence rates among those aged 15-49 increased significantly between 1990 and 2016, followed by a slight decrease until 2019. Among individuals aged 50-69, rates remained stable or increased slightly from 2006 to 2019, while rates in those aged 70 and older have been declining since 2000. In Europe and Central Asia, incidence rates across all age groups have remained relatively stable since the mid-2000s(Murphy and Zaki, 2024). Our study produced similar results. In reports with age-specific information, 67.2% of informants in FAERS and 95.0% in JADER were over 18 and 20 years of age, respectively, aligning with the epidemiological characteristics of CRC. Additionally, a higher proportion of reports were observed in males in both FAERS (45.6% vs 33.7%) and JADER (60.4% vs 36.4%). In their 2018 report, the World Cancer Research Fund and the American Institute for Cancer Research identified various risk factors for CRC, including smoking, inflammatory bowel disease, physical inactivity, dietary habits, alcohol consumption, obesity, non-steroidal anti-inflammatory drug use, and height. Among these, males may have higher levels of exposure to certain risk factors, contributing to the sex disparity in CRC incidence (Keum and Giovannucci, 2019). Given that demographic factors can influence the reporting of ADEs, understanding these baseline differences is crucial for improving the accuracy of our findings.

### 4.2 SOC for which both databases satisfy the thresholds

#### 4.2.1 Blood and lymphatic system disorders

##### 4.2.1.1 Myelosuppression

A major dose-limiting adverse effect of irinotecan therapy is myelosuppression(Chau et al., 2007). In our study, irinotecan was associated with various forms of myelosuppression, including neutropenia, thrombocytopenia, and leukopenia. Studies have shown varying rates of myelosuppression across different populations. For instance, in a phase II study of Korean small cell lung cancer patients, 89% developed neutropenia, and 59% had anemia when irinotecan was combined with cisplatin (Han et al., 2005). A French study observed transient neutropenia in 80% of patients treated with irinotecan (at a dose of 350 mg/m^2^ every 3 weeks), with severe neutropenia in 47% of total cases (Bleiberg and Cvitkovic, 1996). Similarly, 85% of participants in a U.S. study of docetaxel and irinotecan developed grade 3/4 neutropenia (Adjei et al., 2000). Regional differences in hematological toxicity incidence may be due to variations in dosing, medications, and baseline characteristics (Bleiberg and Cvitkovic, 1996; Wiseman and Markham, 1996).

Although irinotecan-induced myelosuppression is typically reversible, non-cumulative, and short-lived (Glimelius, 2005), febrile neutropenic patients at high risk for infections require prompt treatment with hematopoietic colony-stimulating factors and antibiotics (Adjei et al., 2000; Jansman et al., 2001). Furthermore, the *UGT1A128* polymorphism has been strongly associated with irinotecan-induced neutropenia, and dose adjustments may help reduce hematological toxicity in affected patients (Kweekel et al., 2008; Hulshof et al., 2020; Su et al., 2023).

#### 4.2.2 gastrointestinal disorder

##### 4.2.2.1 Diarrhoea

Another major dose-limiting adverse effect of irinotecan therapy is diarrhea, particularly unpredictable and severe forms(Xu et al., 2024). Diarrhea can lead to treatment delays, electrolyte imbalances, hemodynamic disturbances, and even life-threatening sepsis (Tang et al., 2019). Diarrhea induced by irinotecan is classified into early- and late-onset types. Early-onset diarrhea occurs within 24 h of administration, typically accompanied by vomiting, sweating, and abdominal cramps. It is linked to acetylcholinesterase inhibition, which increases cholinergic activity and disrupts intestinal function. Clinical studies show that early-onset diarrhea affects up to 85% of patients receiving irinotecan (Rougier et al., 1997). Anticholinergic drugs, such as atropine or scopolamine, can mitigate this type of diarrhea (Xu et al., 2024).Late-onset diarrhea, occurring more than 24 h post-administration, is often more severe and prolonged. This type is caused by the overexposure of intestines to SN-38, irinotecan’s active metabolite, leading to mucosal damage. Pro-inflammatory cytokines and prostaglandins may also contribute (Glimelius, 2005; Yang et al., 2005; Tang et al., 2019). The incidence of late-onset diarrhea ranges from 60% to 87% in various clinical trials (Bleiberg and Cvitkovic, 1996; Conti et al., 1996), with a slightly lower rate in Japanese studies due to lower dosing regimens (Shimada et al., 1993).

In our study, diarrhea was among the most frequently reported adverse events in both FAERS [n = 1,728, ROR 4.77 (95% CI: 4.55–5.01), PRR 4.59, EBGM05 4.40, IC025 2.12] and JADER [n = 1,053, ROR 9.78 (95% CI: 9.20–10.41), PRR 9.08, EBGM05 7.94, IC025 1.41], with strong signal values. These results are consistent with clinical trial data. Prophylactic high-dose loperamide may reduce the incidence of severe late-onset diarrhea (Yang et al., 2005).

#### 4.2.3 Metabolism and nutrition disorders

##### 4.2.3.1 Cholinergic syndrome

Irinotecan treatment commonly induces acute side effects, including bradycardia, hypersalivation, abdominal cramps, diarrhea, sweating, and visual disturbances, which are consistent with cholinergic syndrome (Gandia et al., 1993; Rowinsky et al., 1994; Pitot et al., 2000). These symptoms typically resolve within hours after infusion but can significantly impact the patient’s quality of life. The cholinergic effects are believed to result from irinotecan’s inhibition of acetylcholine breakdown, leading to excessive muscarinic receptor stimulation (Kanbayashi et al., 2018). A Japanese retrospective study of 179 irinotecan-treated patients found cholinergic syndrome in 51, with sweating being the most common symptom, followed by diarrhea and abdominal pain (Tsuboya et al., 2019). Furthermore, a case study reported repeated bradycardia in a patient with recurrent colorectal cancer after irinotecan administration (Miya et al., 1998). Logistic regression analysis identified female sex and irinotecan dose as key predictors of cholinergic syndrome (Kanbayashi et al., 2018). Most studies on this syndrome come from Japan, possibly due to differences in body composition between Western and Japanese populations.

To manage this syndrome, dose reduction and prophylactic anticholinergic therapy, such as atropine or butylscopolamine, have proven effective (Kanbayashi et al., 2018; Iihara et al., 2019; Tsuboya et al., 2019). Over 90% of patients receiving prophylactic anticholinergic therapy did not develop cholinergic syndrome, emphasizing its clinical significance (Tsavaris et al., 2003; Cheng et al., 2015; Iihara et al., 2019; Uchiyama et al., 2021).

### 4.3 SOCs that meet thresholds in FAERS only

#### 4.3.1 Hepatobiliary disorders

##### 4.3.1.1 Steatohepatitis

Steatohepatitis emerged as an unexpected signal in the FAERS database [n = 26, ROR 85.60 (95% CI: 57.66–127.10), PRR 85.54, EBGM05 58.21, IC025 5.77]. Evidence suggests that irinotecan treatment may induce various forms of liver damage, including steatohepatitis, particularly in patients with colorectal cancer liver metastases (CRCLM) (Williams et al., 2011; Yahagi et al., 2017; Desjardin et al., 2019). A meta-analysis found that one in 12 patients undergoing hepatic resection for CRCLM following irinotecan treatment eventually developed steatohepatitis(Robinson et al., 2012). Pawlik et al. reported a higher incidence of moderate to severe steatohepatitis in CRCLM patients receiving neoadjuvant irinotecan (Pawlik et al., 2007), and Vauthey et al. found that irinotecan preoperative chemotherapy increased steatohepatitis prevalence and postoperative mortality (Vauthey et al., 2006). Irinotecan induces steatohepatitis through multiple mechanisms, including inhibition of fatty acid oxidation and direct cytotoxicity to mitochondria (Corcelle et al., 2007; Labbe et al., 2008; Wu and Cederbaum, 2013). It also alkalinizes hepatocyte lysosomal pH, leading to lipid accumulation, fatty acid synthesis, and coenzyme A sequestration (Schumacher and Guo, 2015; Mahli et al., 2018). Additionally, irinotecan-induced steatohepatitis is often accompanied by neutrophil infiltration, elevated reactive oxygen species, and increased pro-inflammatory cytokines (Marcolino Assis-Júnior et al., 2017), indicating hepatocellular dysfunction and inflammation (Gomez et al., 2007; Makowiec et al., 2011; Celik et al., 2015; Meunier and Larrey, 2020). Irinotecan-induced steatohepatitis may increase the risk of fibrosis, cirrhosis, and liver failure, particularly in patients with obesity, high BMI, or diabetes, necessitating careful evaluation and monitoring during treatment (Han et al., 2021). Several preclinical studies have identified potential therapeutic agents, such as silymarin, pioglitazone, and sorafenib, which may mitigate irinotecan-induced steatohepatitis (Han et al., 2021).

##### 4.3.1.2 Elevated aminotransferase

Irinotecan-induced hepatotoxicity, including elevated transaminases, is a well-documented adverse effect (Ando et al., 1997). Our findings identified positive signals for elevated liver enzymes In a phase II study of irinotecan (350 mg/m^2^) and raltitrexed (3 mg/m^2^) for advanced CRC, six patients (7%) experienced transaminase elevation, resulting in treatment delays (Feliu et al., 2004). A phase II trial of neoadjuvant irinotecan, capecitabine, and radiotherapy for rectal cancer found transaminase elevation in 19% of patients, with one (3%) developing hyperbilirubinemia (Willeke et al., 2007). In pediatric neuroblastoma patients, 28% had alanine aminotransferase elevation following irinotecan-based treatment (Mody et al., 2017). A phase I trial of sorafenib and irinotecan for hepatoblastoma reported a 50% incidence of transaminase elevation (Keino et al., 2020). Variability in the incidence of transaminase elevation may reflect differences in drug combinations, irinotecan dosage, and study sample sizes. Preclinical studies showed transaminase levels increased 5-11 times the upper limit in rats after irinotecan administration, linked to elevated SN-38 concentrations (de Jong et al., 2007). This highlights the cumulative risk of hepatotoxicity when irinotecan is co-administered with other agents. Regular liver function monitoring and irinotecan dose adjustments are recommended in clinical practice.

### 4.4 SOCs that meet thresholds in JADER only

#### 4.4.1 Fatigue

In the JADER database, the signal strength for fatigue (n = 73) was ROR 4.69 (95% CI: 3.71–5.93), PRR 4.67, EBGM05 3.57, and IC025 0.51. In a global phase III trial of 117 metastatic pancreatic ductal adenocarcinoma patients, 14% (n = 16) experienced grade 3 or four fatigue after nanoliposomal irinotecan combined with fluorouracil and folinic acid (Wang-Gillam et al., 2016). A phase II trial for recurrent glioblastoma patients treated with irinotecan and bevacizumab found 75.9% experienced fatigue, with 8.9% having grade 3 or higher (Friedman et al., 2009). In an Asian phase II trial, 13% of Korean patients (n = 88) receiving irinotecan, fluorouracil, and calcium folinate liposomes had grade 3-4 fatigue (Yoo et al., 2021). Japanese studies reported grade 3-4 fatigue in 2.5%–33% of patients treated with irinotecan alone or in combination (Yoshino et al., 2007; Oshita et al., 2013; Kawakami et al., 2016; Osumi et al., 2018; Kawamoto et al., 2022). In contrast, the FAERS database did not show fatigue as a positive signal (n = 444, ROR 0.97 [95% CI: 0.88–1.06], PRR 0.97, EBGM05 0.89, IC025–0.19), likely because fatigue occurrence in FAERS was not disproportionately linked to irinotecan compared to other drugs in the database.

#### 4.4.2 Decreased appetite

Our study found a significant number of cases of decreased appetite with corresponding positive signal values (n = 494, ROR 5.19 [95% CI: 4.74–5.69], PRR 5.05, EBGM05 4.44, IC025 0.62). In a Japanese study, 16.1% of patients with malignant pleural mesothelioma experienced appetite loss after receiving irinotecan (60 mg/m^2^) and gemcitabine (800 mg/m^2^) (Koda et al., 2021). Similarly, a trial of 261 patients with advanced metastatic colon cancer in Japan reported a 15.3% incidence of appetite loss following irinotecan-based treatment (Watanabe et al., 2023), highlighting a strong association between irinotecan and appetite loss. In another Japanese study, 74.2% of patients treated with FOLFIRI and abciximab for mCRC experienced some degree of appetite loss, with 12.9% reporting grade 3 or higher (Denda et al., 2019). It is important to consider that appetite loss can also result from underlying diseases, psychological stress, tumor-related factors, and inflammatory responses. A global phase III study found that decreased appetite in metastatic pancreatic adenocarcinoma patients treated with gemcitabine and irinotecan was linked to poorer survival outcomes (Macarulla Mercadé et al., 2020). Therefore, patients experiencing appetite loss should receive appropriate nutritional support, which may improve their prognosis.

### 4.5 Other unexpected signals

#### 4.5.1 The second primary malignancy

Second primary malignancy (SPM) is defined as a distinct pathological diagnosis that can originate from the same or a different site as the first primary malignancy (Voûte, 2000). Chemotherapy is known to increase the risk of both hematological and solid malignancies, especially with the use of platinum-based drugs and alkylating agents(Geng et al., 2023). Notably, the risk of SPM is higher when chemotherapy is administered in combination regimens compared to single-agent therapies (Swerdlow et al., 2011). The development of SPMs has been linked to the formation of catechols during the metabolism of chemotherapeutic agents (Hartmann and Lipp, 2006). Additionally, genetic susceptibility plays a significant role in the occurrence of SPMs (Kony et al., 1997). Our study identified SPM as a positive signal in both the FAERS and JADER databases. Irinotecan may inhibit topoisomerase I through its active metabolite SN-38, leading to impaired DNA replication and transcription, which in turn causes DNA damage and genomic instability. This increases the mutation rate, thereby elevating the risk of SPM (Mei et al., 2020). In addition, the hematotoxicity of irinotecan may affect the generation and function of immune cells. This immunosuppressive state may reduce the body’s ability to surveil and eliminate newly formed tumor cells, thereby increasing the likelihood of SPM occurrence (Frese-Schaper et al., 2014; Fang et al., 2016). Finally, in clinical practice, the frequent combination of irinotecan with other chemotherapeutic agents, including high-risk drugs such as cisplatin, also increases the risk of SPM. This necessitates closely monitoring the genomic health and immune function of patients during irinotecan treatment. It is important to emphasize that the development of a second primary tumor may occur long after the initial chemotherapy treatment (Li et al., 2010; Zhai et al., 2018). Therefore, long-term and regular tumor screening and early detection can help identify and address these newly developed malignancies in a timely manner, ultimately improving patients' survival rates and quality of life.

#### 4.5.2 Hyperammonaemia

Hyperammonaemia is a clinical condition characterized by elevated serum ammonia levels, presenting with symptoms such as hypotonia, seizures, vomiting, and abnormal neurological changes, including coma. If left untreated, hyperammonaemia can cause irreversible damage to the developing brain, leading to postural and cognitive deficits (e.g., intellectual disability), seizures, and cerebral palsy, with potentially fatal outcomes (Auron and Brophy, 2012). In our study, hyperammonaemic encephalopathy and hyperammonaemia emerged as unexpected signals in FAERS and JADER, respectively. Although there have been a few previous reports of hyperammonaemia associated with irinotecan administration, its occurrence remains relatively rare. For instance, in a clinical trial conducted in Japan involving 12 patients with advanced gastric cancer, one patient developed severe hyperammonaemia after receiving combination therapy that included irinotecan (Tsuji et al., 2012). Signal detection at the SOC level in our study revealed positive signals for irinotecan, both at the hepatic and metabolic levels. Given that the hepatic urea cycle is the primary pathway for ammonia detoxification, liver dysfunction is often linked to impaired ammonia regulation, leading to hyperammonaemia (Varga et al., 2018). Additionally, it is well established that various metabolic disorders can result in elevated ammonia levels (Singh et al., 2018). During chemotherapy, patients may experience decreased appetite and insufficient protein intake, which can lead to increased muscle catabolism and subsequently elevated ammonia production. Whether irinotecan directly impacts ammonia metabolism by reducing ammonia elimination, aside from the potential mechanisms mentioned above, requires further investigation (Krauss et al., 2012; Boilève et al., 2019b). Monitoring ammonia levels and liver function parameters in patients receiving irinotecan therapy is critically important. For cases of irinotecan-associated hyperammonaemia, discontinuation of the suspected medication, along with increased hydration and adherence to a low-protein diet, is essential for managing the condition (Häberle, 2011; Auron and Brophy, 2012; Boilève et al., 2019b).

#### 4.5.3 Hiccup

Hiccups are myoclonic jerks that primarily affect the diaphragm. While not life-threatening, hiccups can negatively impact daily activities, speech, eating, sleep, and mood. In cancer patients, persistent hiccups may lead to weight loss, fatigue, exhaustion, and increased pain intensity. Prolonged or chronic hiccups can result in depression, reduced oral intake, insomnia, and malnutrition(Ergen et al., 2021). Previous studies have suggested that hiccups may be an adverse reaction to certain medications used during chemotherapy (Errante et al., 2005). For example, Lee et al. successfully reduced hiccups in a patient by switching from dexamethasone to methylprednisolone in a dexamethasone-containing chemotherapy regimen (Lee et al., 2013).

According to a study by Takiguchi Y. et al., hiccups were reported in 49 out of 16,518 patients after treatment with irinotecan. Additionally, Hosoya R. et al. identified irinotecan as a risk factor for hiccups through multiple logistic regression analysis using the JADER database (Takiguchi et al., 2002). This aligns with our findings, as both FAERS and JADER studies showed that hiccups were a positive signal associated with irinotecan use. The potential mechanisms of irinotecan-induced hiccups are as follows: 1. Neurotoxicity: Irinotecan and its active metabolite SN-38 may exert direct effects on the central nervous system, particularly the medullary hiccup center, thereby triggering the hiccup reflex (Zhu et al., 2024; Marjoncu and Jones, 2025). 2. Gastrointestinal irritation: Irinotecan has known irritant effects on the gastrointestinal tract, which can lead to symptoms such as nausea, vomiting, and diarrhea. These gastrointestinal disturbances may indirectly contribute to the onset of hiccups (Sakakibara, 2023). 3. Electrolyte disturbances: Chemotherapeutic agents may cause electrolyte imbalances, such as hyponatremia or hypokalemia, which can impair neuromuscular function and precipitate hiccups (Tanneau et al., 1993; Pandey et al., 2022). Baclofen has shown promise as a treatment option for managing hiccups that occur during chemotherapy (Smith and Busracamwongs, 2003). Timely implementation of preventive and therapeutic strategies—such as administration of anti-hiccup agents, adjustment of the chemotherapeutic regimen, or provision of symptomatic support—may effectively alleviate this adverse effect and significantly improve patients' quality of life.

#### 4.5.4 Hepatic failure

In the clinical priority score analysis of this study, hepatic failure (n = 56; ROR: 3.09, PRR: 3.09, EBGM05: 2.48, IC025: 1.24) emerged as the only high clinical priority signal (total score: 6) and represents an unexpected signal. The findings were further supported by the sensitivity analysis, indicating a potentially strong association between irinotecan use and hepatic failure, warranting heightened clinical attention. A case report described a patient with pancreatic cancer who died from irinotecan-induced steatohepatitis and subsequent hepatic failure (Araz et al., 2021). Furthermore, a systematic review of hepatic injury following chemotherapy in colorectal cancer patients found that, compared to oxaliplatin, irinotecan may significantly increase the risk of hepatic failure and postoperative mortality (Baumgaertner et al., 2010). Mechanistically, irinotecan is metabolized *in vivo* to its active metabolite SN-38, which possesses hepatotoxic potential. SN-38 may impair bile secretion, leading to cholestasis and hepatocellular injury (Bansal et al., 2008). Additionally, it may induce apoptosis of hepatocytes, reducing the population of functional liver cells and consequently compromising hepatic metabolic and detoxification capacity (Suzuki and Kato, 1996; Liu et al., 2022). In clinical practice, understanding the underlying mechanisms of irinotecan-associated hepatic injury is critical for the early identification, prevention, and management of potential hepatic failure. It is recommended to assess patients for metabolic syndrome-related risk factors—such as diabetes, hypertension, hyperlipidemia, and obesity—prior to initiating irinotecan therapy. During treatment, close monitoring of liver function parameters is essential to promptly detect and address potential hepatic adverse events, thereby ensuring patient safety and optimizing therapeutic outcomes.

#### 4.5.5 Neuropathy peripheral

Chemotherapy-induced peripheral neuropathy is one of the common adverse events associated with the FOLFIRINOX regimen. Previous studies have reported that the incidence of grade 3–4 peripheral neuropathy following FOLFIRINOX treatment ranges from 0% to 25% (Sugimoto et al., 2021). In a phase II clinical trial involving 32 patients with chemotherapy-refractory mCRC, 53.4% of patients developed varying degrees of peripheral neuropathy after receiving FOLFIRINOX in combination with bevacizumab (Bellio et al., 2025). A similar incidence rate (56%) was also reported by Faivre S et al. (Faivre et al., 2008). In our study, peripheral neuropathy was similarly identified as a positive signal in both the FAERS and JADER pharmacovigilance databases, and this signal was further supported by sensitivity analysis. Although the precise mechanisms underlying irinotecan-induced peripheral neuropathy remain incompletely understood, current evidence suggests that irinotecan and its active metabolite may exert direct neurotoxic effects on the peripheral nervous system. These effects can lead to axonal injury of neurons, disrupt neural signal transmission, and result in sensory abnormalities (McQuade et al., 2017). Furthermore, irinotecan has been shown to increase oxidative stress in neural tissues, which may contribute to neuronal damage and exacerbate peripheral neurotoxicity (He et al., 2023). Therefore, in clinical practice, clinicians should be vigilant regarding irinotecan-associated peripheral neurotoxicity and adopt individualized management strategies—such as dose adjustment, prolongation of dosing intervals, or the use of neuroprotective agents—to minimize neurological adverse effects and optimize both treatment adherence and patient quality of life.

### 4.6 TTO analysis

Understanding the time of onset time of ADEs helps clinical providers to better manage ADE events. In our study, we analyzed the TTO of ADEs reported in FAERS and JADER, finding that the median TTO was 28 days (IQR: 9-76) in FAERS and 17 days (IQR: 9-57) in JADER. Consistently, the shape parameter β from the Weibull distribution test showed an upper limit of the 95% confidence interval (CI) less than one (early decay type) in both cohorts, indicating a decreasing probability of ADEs over time. A clinical study by Furuse J. et al., based on a Japanese population, reported median TTOs for neutropenia, diarrhea, hepatic dysfunction, and anorexia of 21, 9, 22, and 4 days, respectively (Furuse et al., 2023). Additionally, a study by Okunaka M., also based on JADER, showed that the median TTO for diarrhea ranged from 8 to 14 days with irinotecan monotherapy or combination therapy (Okunaka et al., 2021). These findings are generally consistent with our observations in JADER. Furthermore, evidence from Europe suggests that irinotecan-induced late-onset diarrhea and neutropenia typically occur around 6 and 8 days after dosing (Bleiberg and Cvitkovic, 1996). Interestingly, in a European study, the median TTO was 5 days for patients treated with 350 mg/m^2^ irinotecan every 3 weeks (Yang et al., 2005), while in a U.S. trial, patients treated weekly with 125 mg/m^2^ for 4 weeks had a median TTO of 11 days (Hecht, 1998). We hypothesize that these differences may be related to various factors, including the number of reports, ethnicity, dosage administered, comorbidities, and the underlying disease state (Wiseman and Markham, 1996).

### 4.7 Limitations

Nonetheless, it is crucial to recognize the limitations of our study. Firstly, in this study, the FAERS and JADER databases represent populations primarily from the United States and Japan, respectively, and there may be differences between these populations in terms of patient age, gender, ethnicity, lifestyle, and other health factors. Additionally, the comorbidities and clinical conditions of the reporters may differ across regions, which could influence the incidence of ADEs. Therefore, when interpreting the results, especially when generalizing the conclusions to different countries and regions, it is essential to account for potential confounding factors arising from these demographic differences (He et al., 2025). Secondly, significant differences may also exist in the reporting practices between FAERS and JADER. In the FAERS database, patients can report ADEs through multiple channels, including pharmaceutical company websites or FDA platforms. In contrast, in Japan, patients generally need to report ADEs through their physicians or pharmacists, which introduces a difference in reporting pathways and data collection methods. This difference may lead to disparities in the total number of cases between the two databases, thereby affecting the comparison of data (Zou et al., 2024). Thirdly, SRS databases inherently have limitations such as underreporting, duplicate reporting, and reporting bias. Underreporting can lead to an underestimation of the incidence of certain ADEs (Ge et al., 2025). Additionally, although FDA-recommended data cleaning and deduplication processes have been implemented, duplicate reports may still exist, potentially leading to an overestimation of the signal strength for certain adverse events (Liu et al., 2025). Reporting bias can arise from factors such as the willingness of healthcare professionals to report, patients' ability to self-report, and the criteria for identifying ADEs. In some cases, more severe ADEs may be more likely to be reported, while mild ADEs may be overlooked or underreported, which could result in the occurrence rate of severe events being higher than the actual incidence (Jiang et al., 2025). Fourthly, although we selected only the primary suspect drug role codes for ADEs in this study and conducted sensitivity analysis to exclude concomitant medications, potential confounding variables, such as dosage, duration of use, and concomitant medication, may still affect the accuracy of our results (Wang et al., 2024; Wei et al., 2024). For example, certain chemotherapy drugs may increase the risk of adverse reactions to irinotecan or interact with it, leading to varying clinical outcomes, which makes the results more complex. Fifthly, signal detection in pharmacovigilance studies primarily reveals statistical associations, aiming to estimate the strength of a signal rather than establish causality. To confirm whether these statistical associations are causal, further prospective clinical studies are required (Zou et al., 2024). Despite the limitations outlined above, the cross-validation method using the FAERS and JADER databases still provides valuable insights and guidance for post-marketing safety monitoring of irinotecan and the detection of rare signals.

## 5 Conclusion

In conclusion, this study thoroughly explored ADEs associated with irinotecan and assessed them using disproportionality analysis of real-world data from the FAERS and JADER databases. The signals identified in this study that consistent with those listed in the drug label include diarrhea, neutropenia, thrombocytopenia, stomatitis, and enteritis. Additionally, we identified several unexpected signals, such as palmar-plantar erythrodysaesthesia syndrome, hyperammonaemia, steatohepatitis, second primary malignancy, and hiccups in the FAERS database, as well as myelodysplastic syndrome, cholinergic syndrome, peripheral sensory neuropathy, paronychia, and acne in the JADER database. Furthermore, we identified hepatic failure in the FAERS database as a signal with a high clinical priority score. Finally, we analyzed the onset time of these ADEs to provide healthcare providers with useful reference information. These findings offer essential safety considerations for the clinical use of irinotecan and highlight the importance of careful patient monitoring. However, given the limitations of this study, it is crucial to conduct prospective clinical trials and collect long-term data to validate these results.

## Data Availability

The original contributions presented in the study are included in the article/Supplementary Material, further inquiries can be directed to the corresponding authors.

## Supplementary material

SUPPLEMENTARY FIGURE S1 Bar chart comparing the composition of ADE reports at the SOC level between the two databases. FAERS: The U.S. FDA Adverse Event Reporting System; JADER: Japanese Adverse Drug Event Report. SOC: system organ class.

SUPPLEMENTARY FIGURE S2 The overlap of positive signals identified from the FAERS and JADER databases is illustrated using a Venn diagram. The PTs marked in white represent unexpected signals identified in both databases, while the PTs marked in black represent signals that are consistent with the drug’s label, identified in both databases. FAERS: The U.S. FDA Adverse Event Reporting System; JADER, Japanese Adverse Drug Event Report.

## References

[B1] Adjei A. A. Klein C. E. Kastrissios H. Goldberg R. M. Alberts S. R. Pitot H. C. (2000). Phase I and pharmacokinetic study of irinotecan and docetaxel in patients with advanced solid tumors: preliminary evidence of clinical activity. J. Clin. Oncol. official J. Am. Soc. Clin. Oncol. 18 (5), 1116–1123. 10.1200/JCO.2000.18.5.1116 10694565

[B2] Algeciras-Schimnich A. O'kane D. J. Snozek C. L. H. (2008). Pharmacogenomics of tamoxifen and irinotecan therapies. Clin. Laboratory Med. 28 (4), 553–567. 10.1016/j.cll.2008.05.004 19059062

[B3] Alimonti A. Gelibter A. Pavese I. Satta F. Cognetti F. Ferretti G. (2004). New approaches to prevent intestinal toxicity of irinotecan-based regimens. Cancer Treat. Rev. 30 (6), 555–562. 10.1016/j.ctrv.2004.05.002 15325035

[B4] Ando M. Eguchi K. Shinkai T. Tamura T. Ohe Y. Yamamoto N. (1997). Phase I study of sequentially administered topoisomerase I inhibitor (irinotecan) and topoisomerase II inhibitor (etoposide) for metastatic non-small-cell lung cancer. Br. J. Cancer 76 (11), 1494–1499. 10.1038/bjc.1997.584 9400948 PMC2228187

[B5] Araz M. Kilinc F. Kerimoglu U. Keskin M. Kucukkartallar T. (2021). Irinotecan-induced NASH and liver failure. Clin. Res. Hepatology Gastroenterology 45 (3), 101606. 10.1016/j.clinre.2020.101606 33446474

[B6] Auron A. Brophy P. D. (2012). Hyperammonemia in review: pathophysiology, diagnosis, and treatment. Pediatr. Nephrol. 27 (2), 207–222. 10.1007/s00467-011-1838-5 21431427

[B7] Bailly C. (2019). Irinotecan: 25 years of cancer treatment. Pharmacol. Res. 148, 104398. 10.1016/j.phrs.2019.104398 31415916

[B8] Bansal T. Awasthi A. Jaggi M. Khar R. K. Talegaonkar S. (2008). Pre-clinical evidence for altered absorption and biliary excretion of irinotecan (CPT-11) in combination with quercetin: possible contribution of P-glycoprotein. Life Sci. 83 (7-8), 250–259. 10.1016/j.lfs.2008.06.007 18619980

[B9] Bate A. Lindquist M. Edwards I. R. Olsson S. Orre R. Lansner A. (1998). A Bayesian neural network method for adverse drug reaction signal generation. Eur. J. Clin. Pharmacol. 54 (4), 315–321. 10.1007/s002280050466 9696956

[B10] Baumgaertner I. Ratziu V. Vaillant J. C. Hannoun L. Poynard T. André T. (2010). Hepatotoxicity of metastatic colorectal cancer chemotherapy: systematic review. Bull. Du. Cancer 97 (5), 559–569. 10.1684/bdc.2010.1049 20167564

[B11] Bellio H. Roussot N. Bertaut A. Hervieu A. Zanetta S. Tharin Z. (2025). FOLFIRINOX-3 plus bevacizumab (bFOLFIRINOX3) in chemo-refractory metastatic colorectal cancer: a multicenter phase II trial. Future Oncol. Lond. Engl. 21 (6), 699–706. 10.1080/14796694.2025.2461446 PMC11881852 39913183

[B12] Bleiberg H. Cvitkovic E. (1996). Characterisation and clinical management of CPT-11 (irinotecan)-induced adverse events: the European perspective. Eur. J. Cancer Oxford, Engl. 1990 32A (Suppl. 3), S18–S23. 10.1016/0959-8049(96)00293-6 8943661

[B13] Boilève A. Jozwiak M. Malka D. Boige V. Le Roy F. Paques M. (2019a). Vision loss after chemotherapy: an irinotecan-induced retinopathy. Eur. J. Cancer Oxford, Engl. 1990 112, 80–82. 10.1016/j.ejca.2019.02.015 30947025

[B14] Boilève A. Wicker C. Verret B. Leroy F. Malka D. Jozwiak M. (2019b). 5-Fluorouracil rechallenge after 5-fluorouracil-induced hyperammonemic encephalopathy. Anti-cancer Drugs 30 (3), 313–317. 10.1097/CAD.0000000000000730 30531368

[B15] Caster O. Aoki Y. Gattepaille L. M. Grundmark B. (2020). Disproportionality analysis for pharmacovigilance signal detection in small databases or Subsets: Recommendations for limiting false-positive associations. Drug Saf. 43 (5), 479–487. 10.1007/s40264-020-00911-w 32008183 PMC7165139

[B16] Cecco S. Puligheddu S. Fusaroli M. Gerratana L. Yan M. Zamagni C. (2024). Emerging toxicities of Antibody-drug Conjugates for breast cancer: clinical Prioritization of adverse events from the FDA adverse event reporting system. Target. Oncol. 19 (3), 435–445. 10.1007/s11523-024-01058-9 38696126 PMC11111510

[B17] Celik S. Kartal K. Ozseker H. Hayran M. Hamaloglu E. (2015). Hepatoprotective effect of pioglitazone in cases of chemotherapy induced steatohepatitis. Chir. Buchar. Rom. 1990 110 (1), 49–55. 25800316

[B18] Chau I. Cunningham D. Hickish T. Massey A. Higgins L. Osborne R. (2007). Gefitinib and irinotecan in patients with fluoropyrimidine-refractory, irinotecan-naive advanced colorectal cancer: a phase I-II study. Ann. Oncol. Official J. Eur. Soc. For Med. Oncol. 18 (4), 730–737. 10.1093/annonc/mdl481 17237473

[B19] Chen Z. Jiang L. (2019). The clinical application of fruquintinib on colorectal cancer. Expert Rev. Clin. Pharmacol. 12 (8), 713–721. 10.1080/17512433.2019.1630272 31177854

[B20] Cheng C. Lau J. E. Earl M. A. (2015). Use of atropine-diphenoxylate compared with hyoscyamine to decrease rates of irinotecan-related cholinergic syndrome. J. Community Support. Oncol. 13 (1), 3–7. 10.12788/jcso.0099 25839059

[B21] Conroy T. Bosset J.-F. Etienne P.-L. Rio E. François É. Mesgouez-Nebout N. (2021). Neoadjuvant chemotherapy with FOLFIRINOX and preoperative chemoradiotherapy for patients with locally advanced rectal cancer (UNICANCER-PRODIGE 23): a multicentre, randomised, open-label, phase 3 trial. Lancet. Oncol. 22 (5), 702–715. 10.1016/S1470-2045(21)00079-6 33862000

[B22] Conroy T. Desseigne F. Ychou M. Bouché O. Guimbaud R. Bécouarn Y. (2011). FOLFIRINOX versus gemcitabine for metastatic pancreatic cancer. N. Engl. J. Med. 364 (19), 1817–1825. 10.1056/NEJMoa1011923 21561347

[B23] Conti J. A. Kemeny N. E. Saltz L. B. Huang Y. Tong W. P. Chou T. C. (1996). Irinotecan is an active agent in untreated patients with metastatic colorectal cancer. J. Clin. Oncol. Official J. Am. Soc. Clin. Oncol. 14 (3), 709–715. 10.1200/JCO.1996.14.3.709 8622015

[B24] Corcelle E. Djerbi N. Mari M. Nebout M. Fiorini C. Fénichel P. (2007). Control of the autophagy maturation step by the MAPK ERK and p38: lessons from environmental carcinogens. Autophagy 3 (1), 57–59. 10.4161/auto.3424 17102581

[B25] Cui Z. Cheng F. Wang L. Zou F. Pan R. Tian Y. (2023). A pharmacovigilance study of etoposide in the FDA adverse event reporting system (FAERS) database, what does the real world say? Front. Pharmacol. 14, 1259908. 10.3389/fphar.2023.1259908 37954852 PMC10637489

[B26] Cunningham D. Pyrhönen S. James R. D. Punt C. J. Hickish T. F. Heikkila R. (1998). Randomised trial of irinotecan plus supportive care versus supportive care alone after fluorouracil failure for patients with metastatic colorectal cancer. Lancet London, Engl. 352 (9138), 1413–1418. 10.1016/S0140-6736(98)02309-5 9807987

[B27] De Jong F. A. Van Der Bol J. M. Mathijssen R. H. J. Loos W. J. Mathôt R. a. A. Kitzen J. J. E. M. (2007). Irinotecan chemotherapy during valproic acid treatment: pharmacokinetic interaction and hepatotoxicity. Cancer Biol. & Ther. 6 (9), 1368–1374. 10.4161/cbt.6.9.4567 17873515

[B28] De Man F. M. Goey A. K. L. Van Schaik R. H. N. Mathijssen R. H. J. Bins S. (2018). Individualization of irinotecan treatment: a review of pharmacokinetics, Pharmacodynamics, and pharmacogenetics. Clin. Pharmacokinet. 57 (10), 1229–1254. 10.1007/s40262-018-0644-7 29520731 PMC6132501

[B29] Denda T. Sakai D. Hamaguchi T. Sugimoto N. Ura T. Yamazaki K. (2019). Phase II trial of aflibercept with FOLFIRI as a second-line treatment for Japanese patients with metastatic colorectal cancer. Cancer Sci. 110 (3), 1032–1043. 10.1111/cas.13943 30657223 PMC6398889

[B30] Desjardin M. Bonhomme B. Le Bail B. Evrard S. Brouste V. Desolneux G. (2019). Hepatotoxicities induced by neoadjuvant chemotherapy in colorectal cancer liver metastases: distinguishing the True from the false. Oncology 13, 1179554918825450. 10.1177/1179554918825450 30718969 PMC6348554

[B31] Di Desidero T. Antonelli A. Orlandi P. Ferrari S. M. Fioravanti A. Alì G. (2017). Synergistic efficacy of irinotecan and sunitinib combination in preclinical models of anaplastic thyroid cancer. Cancer Lett. 411, 35–43. 10.1016/j.canlet.2017.09.032 28964784 PMC8022336

[B32] Eng C. Yoshino T. Ruíz-García E. Mostafa N. Cann C. G. O'brian B. (2024). Colorectal cancer. Lancet London, Engl. 404 (10449), 294–310. 10.1016/S0140-6736(24)00360-X 38909621

[B33] Ergen M. Arikan F. Fırat Çetin R. (2021). Hiccups in cancer patients receiving chemotherapy: a cross-Sectional study. J. Pain Symptom Manag. 62 (3), e85–e90. 10.1016/j.jpainsymman.2021.02.012 33587996

[B34] Errante D. Bernardi D. Bianco A. Zanatta N. Salvagno L. (2005). Recurrence of exhausting hiccup in a patient treated with chemotherapy for metastatic colon cancer. Gut 54 (10), 1503–1504. 10.1136/gut.2005.071704 16162960 PMC1774708

[B35] Etienne-Grimaldi M.-C. Boyer J.-C. Thomas F. Quaranta S. Picard N. Loriot M.-A. (2015). UGT1A1 genotype and irinotecan therapy: general review and implementation in routine practice. Fundam. & Clin. Pharmacol. 29 (3), 219–237. 10.1111/fcp.12117 25817555

[B36] Evans S. J. Waller P. C. Davis S. (2001). Use of proportional reporting ratios (PRRs) for signal generation from spontaneous adverse drug reaction reports. Pharmacoepidemiol. Drug Saf. 10 (6), 483–486. 10.1002/pds.677 11828828

[B37] Faivre S. Delbaldo C. Boige V. Pautier P. Soria J.-C. Namouni F. (2008). Safety of repeated administrations of ixabepilone given as a 3-hour infusion every other week in combination with irinotecan in patients with advanced malignancies. Eur. J. Cancer Oxford, Engl. 1990 44 (5), 674–682. 10.1016/j.ejca.2008.01.016 18308561

[B38] Fang Z.-Z. Zhang D. Cao Y.-F. Xie C. Lu D. Sun D.-X. (2016). Irinotecan (CPT-11)-induced elevation of bile acids potentiates suppression of IL-10 expression. Toxicol. Appl. Pharmacol. 291, 21–27. 10.1016/j.taap.2015.12.003 26706406 PMC4718832

[B39] Feliu J. Salud A. Escudero P. López-Gómez L. Pericay C. Castañón C. (2004). Irinotecan plus raltitrexed as first-line treatment in advanced colorectal cancer: a phase II study. Br. J. Cancer 90 (8), 1502–1507. 10.1038/sj.bjc.6601713 15083176 PMC2409728

[B40] Frampton J. E. (2020). Liposomal irinotecan: a review in metastatic pancreatic adenocarcinoma. Drugs 80 (10), 1007–1018. 10.1007/s40265-020-01336-6 32557396 PMC7347682

[B41] Frese-Schaper M. Keil A. Steiner S. K. Gugger M. Körner M. Kocher G. J. (2014). Low-dose irinotecan improves advanced lupus nephritis in mice potentially by changing DNA relaxation and anti-double-stranded DNA binding. Arthritis & Rheumatology Hoboken, N.J. 66 (8), 2259–2269. 10.1002/art.38665 24729466

[B42] Friedman H. S. Prados M. D. Wen P. Y. Mikkelsen T. Schiff D. Abrey L. E. (2009). Bevacizumab alone and in combination with irinotecan in recurrent glioblastoma. J. Clin. Oncol. Official J. Am. Soc. Clin. Oncol. 27 (28), 4733–4740. 10.1200/JCO.2008.19.8721 19720927

[B43] Furuse J. Ueno M. Ikeda M. Okusaka T. Teng Z. Furuya M. (2023). Liposomal irinotecan with fluorouracil and leucovorin after gemcitabine-based therapy in Japanese patients with metastatic pancreatic cancer: additional safety analysis of a randomized phase 2 trial. Jpn. J. Clin. Oncol. 53 (2), 130–137. 10.1093/jjco/hyac177 36412114 PMC9885735

[B44] Fusaroli M. Salvo F. Begaud B. Alshammari T. M. Bate A. Battini V. (2024a). The reporting of a disproportionality analysis for drug safety signal detection using individual case safety reports in PharmacoVigilance (READUS-PV): development and Statement. Drug Saf. 47 (6), 575–584. 10.1007/s40264-024-01421-9 38713346 PMC11116242

[B45] Fusaroli M. Salvo F. Begaud B. Alshammari T. M. Bate A. Battini V. (2024b). The REporting of A disproportionality analysis for DrUg safety signal detection using individual case safety reports in PharmacoVigilance (READUS-PV): Explanation and Elaboration. Drug Saf. 47 (6), 585–599. 10.1007/s40264-024-01423-7 38713347 PMC11116264

[B46] Gandia D. Abigerges D. Armand J. P. Chabot G. Da Costa L. De Forni M. (1993). CPT-11-induced cholinergic effects in cancer patients. J. Clin. Oncol. Official J. Am. Soc. Clin. Oncol. 11 (1), 196–197. 10.1200/JCO.1993.11.1.196 8418235

[B47] Ge W. Yan Y. Hu Y. Lin S. Mao P. (2025). Unveiling the safety profile of lecanemab: a comprehensive analysis of adverse events using FDA adverse event reporting system data. J. Alzheimer's Dis. JAD 103 (3), 844–855. 10.1177/13872877241307246 39801129

[B48] Geng F. Liu M. Chen J. Ge Y. Wei S. Li F. (2023). Clinical characteristics of second primary malignancies among first primary malignancy survivors: a single-center study, 2005-2020. Oncol. Lett. 25 (1), 24. 10.3892/ol.2022.13610 36478913 PMC9713803

[B49] Glimelius B. (2005). Benefit-risk assessment of irinotecan in advanced colorectal cancer. Drug Saf. 28 (5), 417–433. 10.2165/00002018-200528050-00005 15853443

[B50] Gomez D. Malik H. Z. Bonney G. K. Wong V. Toogood G. J. Lodge J. P. A. (2007). Steatosis predicts postoperative morbidity following hepatic resection for colorectal metastasis. Br. J. Surg. 94 (11), 1395–1402. 10.1002/bjs.5820 17607707

[B51] Gu J. Qu Y. Shen Y. Zhou Q. Jiang Y. Zhu H. (2024). Comprehensive analysis of adverse events associated with pimavanserin using the FAERS database. J. Affect. Disord. 362, 742–748. 10.1016/j.jad.2024.07.103 39029673

[B52] Häberle J. (2011). Clinical practice: the management of hyperammonemia. Eur. J. Pediatr. 170 (1), 21–34. 10.1007/s00431-010-1369-2 21165747

[B53] Hahn R. Z. Antunes M. V. Verza S. G. Perassolo M. S. Suyenaga E. S. Schwartsmann G. (2019). Pharmacokinetic and pharmacogenetic Markers of irinotecan toxicity. Curr. Med. Chem. 26 (12), 2085–2107. 10.2174/0929867325666180622141101 29932028

[B54] Hai L. Wu J. Pan X. Yin W. Wu Z. (2025). A real-world pharmacovigilance study of FDA adverse event reporting system events for Obeticholic acid. Pharmacoepidemiol. Drug Saf. 34 (1), e70084. 10.1002/pds.70084 39776053

[B55] Han J. Zhang J. Zhang C. (2021). Irinotecan-induced steatohepatitis: current insights. Front. Oncol. 11, 754891. 10.3389/fonc.2021.754891 34707997 PMC8542761

[B56] Han J.-Y. Lee D. H. Lee S. Y. Park C. G. Kim H. Y. Kim E.-A. (2005). A phase II study of dose-intensified weekly concomitant administration of cisplatin and irinotecan in chemonaive patients with extensive-disease small-cell lung cancer. Med. Oncol. N. Lond. Engl. 22 (3), 281–290. 10.1385/MO:22:3:281 16110139

[B57] Hartmann J. T. Lipp H.-P. (2006). Camptothecin and podophyllotoxin derivatives: inhibitors of topoisomerase I and II - mechanisms of action, pharmacokinetics and toxicity profile. Drug Saf. 29 (3), 209–230. 10.2165/00002018-200629030-00005 16524321

[B58] He C.-Z. Qiu Q. Lu S.-J. Xue F.-L. Liu J.-Q. He Y. (2025). Adverse event reporting of faricimab: a disproportionality analysis of FDA adverse event reporting system (FAERS) database. Front. Pharmacol. 16, 1521358. 10.3389/fphar.2025.1521358 40144657 PMC11936923

[B59] He J. Han S. Wang Y. Kang Q. Wang X. Su Y. (2023). Irinotecan cause the side effects on development and adult physiology, and induces intestinal damage via innate immune response and oxidative damage in Drosophila. Biomed. & Pharmacother. = Biomedecine & Pharmacother. 169, 115906. 10.1016/j.biopha.2023.115906 37984304

[B60] Hecht J. R. (1998). Gastrointestinal toxicity or irinotecan. Oncol. Willist. Park, N.Y. 12 (8 Suppl. 6), 72–78. 9726096

[B61] Huisman S. A. De Bruijn P. Ghobadi Moghaddam-Helmantel I. M. Ijzermans J. N. M. Wiemer E. a. C. Mathijssen R. H. J. (2016). Fasting protects against the side effects of irinotecan treatment but does not affect anti-tumour activity in mice. Br. J. Pharmacol. 173 (5), 804–814. 10.1111/bph.13317 26332723 PMC4761088

[B62] Hulshof E. C. Deenen M. J. Guchelaar H.-J. Gelderblom H. (2020). Pre-therapeutic UGT1A1 genotyping to reduce the risk of irinotecan-induced severe toxicity: Ready for prime time. Eur. J. Cancer, 141. 10.1016/j.ejca.2020.09.007 33125947

[B63] Iihara H. Fujii H. Yoshimi C. Kobayashi R. Matsuhashi N. Takahashi T. (2019). Prophylactic effect of scopolamine butylbromide, a competitive antagonist of muscarinic acetylcholine receptor, on irinotecan-related cholinergic syndrome. Cancer Chemother. Pharmacol. 83 (3), 393–398. 10.1007/s00280-018-3736-z 30564875 PMC6394464

[B64] Jansman F. G. Sleijfer D. T. De Graaf J. C. Coenen J. L. Brouwers J. R. (2001). Management of chemotherapy-induced adverse effects in the treatment of colorectal cancer. Drug Saf. 24 (5), 353–367. 10.2165/00002018-200124050-00002 11419562

[B65] Jiang Y. Lu R. Du Z. Shen Y. Zhou Q. Luan P. (2025). The real-world safety assessment of Siponimod: a systematic analysis based on the FAERS database. J. Neurological Sci. 468, 123364. 10.1016/j.jns.2024.123364 39732042

[B66] Kanbayashi Y. Ishikawa T. Kanazawa M. Nakajima Y. Tabuchi Y. Kawano R. (2018). Predictive factors for the development of irinotecan-related cholinergic syndrome using ordered logistic regression analysis. Med. Oncol. N. Lond. Engl. 35 (6), 82. 10.1007/s12032-018-1142-3 29705823

[B67] Kawakami T. Machida N. Yasui H. Kawahira M. Kawai S. Kito Y. (2016). Efficacy and safety of irinotecan monotherapy as third-line treatment for advanced gastric cancer. Cancer Chemother. Pharmacol. 78 (4), 809–814. 10.1007/s00280-016-3138-z 27566700

[B68] Kawamoto Y. Yuki S. Meguro T. Hatanaka K. Uebayashi M. Nakamura M. (2022). Phase II study of continued Trastuzumab plus irinotecan in patients with HER2-positive gastric cancer previously treated with Trastuzumab (HGCSG 1201). Oncol. 27 (5), 340–e374. 10.1093/oncolo/oyab062 PMC9075004 35303078

[B69] Kciuk M. Marciniak B. Kontek R. (2020). Irinotecan-still an important player in cancer chemotherapy: a comprehensive Overview. Int. J. Mol. Sci. 21 (14), 4919. 10.3390/ijms21144919 32664667 PMC7404108

[B70] Keino D. Yokosuka T. Hirose A. Sakurai Y. Nakamura W. Fujita S. (2020). Pilot study of the combination of sorafenib and fractionated irinotecan in pediatric relapse/refractory hepatic cancer (FINEX pilot study). Pediatr. Blood & Cancer 67 (11), e28655. 10.1002/pbc.28655 32798298

[B71] Keum N. Giovannucci E. (2019). Global burden of colorectal cancer: emerging trends, risk factors and prevention strategies. Gastroenterology & Hepatology 16 (12), 713–732. 10.1038/s41575-019-0189-8 31455888

[B72] Kim S. Chen J. Cheng T. Gindulyte A. He J. He S. (2023). PubChem 2023 update. Nucleic Acids Res. 51 (D1), D1373–D1380. 10.1093/nar/gkac956 36305812 PMC9825602

[B73] Kinoshita S. Hosomi K. Yokoyama S. Takada M. (2020). Time-to-onset analysis of amiodarone-associated thyroid dysfunction. J. Clin. Pharm. Ther. 45 (1), 65–71. 10.1111/jcpt.13024 31400296

[B74] Koda Y. Kuribayashi K. Doi H. Kitajima K. Nakajima Y. Ishigaki H. (2021). Irinotecan and gemcitabine as second-line treatment in patients with malignant pleural mesothelioma following platinum plus Pemetrexed chemotherapy: a retrospective study. Oncology 99 (3), 161–168. 10.1159/000510691 33053560

[B75] Kony S. J. De Vathaire F. Chompret A. Shamsaldim A. Grimaud E. Raquin M. A. (1997). Radiation and genetic factors in the risk of second malignant neoplasms after a first cancer in childhood. Lancet London, Engl. 350 (9071), 91–95. 10.1016/S0140-6736(97)01116-1 9228960

[B76] Krauss M. Schaller S. Borchers S. Findeisen R. Lippert J. Kuepfer L. (2012). Integrating cellular metabolism into a multiscale whole-body model. PLoS Comput. Biol. 8 (10), e1002750. 10.1371/journal.pcbi.1002750 23133351 PMC3486908

[B77] Kweekel D. Guchelaar H.-J. Gelderblom H. (2008). Clinical and pharmacogenetic factors associated with irinotecan toxicity. Cancer Treat. Rev. 34 (7), 656–669. 10.1016/j.ctrv.2008.05.002 18558463

[B78] Labbe G. Pessayre D. Fromenty B. (2008). Drug-induced liver injury through mitochondrial dysfunction: mechanisms and detection during preclinical safety studies. Fundam. & Clin. Pharmacol. 22 (4), 335–353. 10.1111/j.1472-8206.2008.00608.x 18705745

[B79] Lam S. W. Guchelaar H. J. Boven E. (2016). The role of pharmacogenetics in capecitabine efficacy and toxicity. Cancer Treat. Rev. 50, 9–22. 10.1016/j.ctrv.2016.08.001 27569869

[B80] Lee G.-W. Oh S. Y. Kang M. H. Kang J. H. Park S. H. Hwang I. G. (2013). Treatment of dexamethasone-induced hiccup in chemotherapy patients by methylprednisolone rotation. Oncol. 18 (11), 1229–1234. 10.1634/theoncologist.2013-0224 PMC3825309 24107973

[B81] Li C. I. Nishi N. Mcdougall J. A. Semmens E. O. Sugiyama H. Soda M. (2010). Relationship between radiation exposure and risk of second primary cancers among atomic bomb survivors. Cancer Res. 70 (18), 7187–7198. 10.1158/0008-5472.CAN-10-0276 20843820 PMC2941904

[B82] Liu B. Ding C. Tang W. Zhang C. Gu Y. Wang Z. (2022). Hepatic ROS Mediated Macrophage activation is responsible for irinotecan induced liver injury. Cells 11 (23), 3791. 10.3390/cells11233791 36497051 PMC9739808

[B83] Liu J.-F. Bai Y.-T. Leng Y.-E. Chang E. Wei Y.-X. Wei W. (2025). Post-marketing safety concerns with luspatercept: a disproportionality analysis of the FDA adverse event reporting system. Expert Opin. Drug Saf., 1–8. 10.1080/14740338.2025.2464071 39912511

[B84] Locke F. L. Go W. Y. Neelapu S. S. (2020). Development and Use of the anti-CD19 Chimeric Antigen receptor T-cell therapy Axicabtagene Ciloleucel in large B-cell lymphoma: a review. JAMA Oncol. 6 (2), 281–290. 10.1001/jamaoncol.2019.3869 31697310 PMC7859915

[B85] Lu B. Lu C. Sun Z. Qu C. Chen J. Hua Z. (2019). Combination of apatinib mesylate and second-line chemotherapy for treating gastroesophageal junction adenocarcinoma. J. Int. Med. Res. 47 (5), 2207–2214. 10.1177/0300060519827191 30991863 PMC6567765

[B86] Lu C.-Y. Yeh Y.-S. Huang C.-W. Ma C.-J. Yu F.-J. Wang J.-Y. (2014). FOLFIRI and regorafenib combination therapy with dose escalation of irinotecan as fourth-line treatment for patients with metastatic colon cancer according to UGT1A1 genotyping. OncoTargets Ther. 7, 2143–2146. 10.2147/OTT.S69774 PMC4246994 25473295

[B87] Macarulla Mercadé T. Chen L.-T. Li C.-P. Siveke J. T. Cunningham D. Bodoky G. (2020). Liposomal irinotecan + 5-FU/LV in metastatic pancreatic cancer: Subgroup Analyses of patient, tumor, and previous treatment characteristics in the Pivotal NAPOLI-1 trial. Pancreas 49 (1), 62–75. 10.1097/MPA.0000000000001455 31856081 PMC6946097

[B88] Mahli A. Saugspier M. Koch A. Sommer J. Dietrich P. Lee S. (2018). ERK activation and autophagy impairment are central mediators of irinotecan-induced steatohepatitis. Gut 67 (4), 746–756. 10.1136/gutjnl-2016-312485 28053052

[B89] Makowiec F. Möhrle S. Neeff H. Drognitz O. Illerhaus G. Opitz O. G. (2011). Chemotherapy, liver injury, and postoperative complications in colorectal liver metastases. J. Gastrointest. Surg. Official J. Soc. For Surg. Alimentary Tract 15 (1), 153–164. 10.1007/s11605-010-1368-7 21061183

[B90] Marcolino Assis-Júnior E. Melo A. T. Pereira V. B. M. Wong D. V. T. Sousa N. R. P. Oliveira C. M. G. (2017). Dual effect of silymarin on experimental non-alcoholic steatohepatitis induced by irinotecan. Toxicol. Appl. Pharmacol. 327, 71–79. 10.1016/j.taap.2017.04.023 28454924

[B91] Marjoncu D. Jones K. (2025). Irinotecan-induced dysarthria and management. J. Oncol. Pharm. Pract., 10781552251324868. 10.1177/10781552251324868 40017267

[B92] Mazhar F. Battini V. Gringeri M. Pozzi M. Mosini G. Marran A. M. N. (2021). The impact of anti-TNFα agents on weight-related changes: new insights from a real-world pharmacovigilance study using the FDA adverse event reporting system (FAERS) database. Expert Opin. Biol. Ther. 21 (9), 1281–1290. 10.1080/14712598.2021.1948529 34191656

[B93] Mcquade R. M. Stojanovska V. Donald E. L. Rahman A. A. Campelj D. G. Abalo R. (2017). Irinotecan-induced gastrointestinal dysfunction is associated with enteric neuropathy, but increased numbers of cholinergic Myenteric neurons. Front. Physiology 8, 391. 10.3389/fphys.2017.00391 PMC5462962 28642718

[B94] Mei C. Lei L. Tan L.-M. Xu X.-J. He B.-M. Luo C. (2020). The role of single strand break repair pathways in cellular responses to camptothecin induced DNA damage. Biomed. & Pharmacother. = Biomedecine & Pharmacother. 125, 109875. 10.1016/j.biopha.2020.109875 32036211

[B95] Meunier L. Larrey D. (2020). Chemotherapy-associated steatohepatitis. Ann. Hepatology 19 (6), 597–601. 10.1016/j.aohep.2019.11.012 32061473

[B96] Miya T. Fujikawa R. Fukushima J. Nogami H. Koshiishi Y. Goya T. (1998). Bradycardia induced by irinotecan: a case report. Jpn. J. Clin. Oncol. 28 (11), 709–711. 10.1093/jjco/28.11.709 9861240

[B97] Mody R. Naranjo A. Van Ryn C. Yu A. L. London W. B. Shulkin B. L. (2017). Irinotecan-temozolomide with temsirolimus or dinutuximab in children with refractory or relapsed neuroblastoma (COG ANBL1221): an open-label, randomised, phase 2 trial. Lancet. Oncol. 18 (7), 946–957. 10.1016/S1470-2045(17)30355-8 28549783 PMC5527694

[B98] Murphy C. C. Zaki T. A. (2024). Changing epidemiology of colorectal cancer - birth cohort effects and emerging risk factors. Gastroenterology & Hepatology 21 (1), 25–34. 10.1038/s41575-023-00841-9 37723270

[B99] Noguchi Y. Tachi T. Teramachi H. (2021). Detection algorithms and attentive points of safety signal using spontaneous reporting systems as a clinical data source. Briefings Bioinforma. 22 (6), bbab347. 10.1093/bib/bbab347 34453158

[B100] Okada N. Niimura T. Zamami Y. Hamano H. Ishida S. Goda M. (2019). Pharmacovigilance evaluation of the relationship between impaired glucose metabolism and BCR-ABL inhibitor use by using an adverse drug event reporting database. Cancer Med. 8 (1), 174–181. 10.1002/cam4.1920 30561126 PMC6346261

[B101] Okunaka M. Kano D. Matsui R. Kawasaki T. Uesawa Y. (2021). Evaluation of the expression profile of irinotecan-induced diarrhea in patients with colorectal cancer. Pharm. Basel, Switz. 14 (4), 377. 10.3390/ph14040377 PMC8073045 33921605

[B102] Oshita F. Sugiura M. Murakami S. Kondo T. Saito H. Yamada K. (2013). Phase II study of nedaplatin and irinotecan in patients with extensive small-cell lung cancer. Cancer Chemother. Pharmacol. 71 (2), 345–350. 10.1007/s00280-012-2011-y 23124649

[B103] Osumi H. Shinozaki E. Mashima T. Wakatsuki T. Suenaga M. Ichimura T. (2018). Phase II trial of biweekly cetuximab and irinotecan as third-line therapy for pretreated KRAS exon 2 wild-type colorectal cancer. Cancer Sci. 109 (8), 2567–2575. 10.1111/cas.13698 29908105 PMC6113428

[B104] Pandey P. K. Pandey D. Andrews R. (2022). Hiccups and acute symptomatic hyponatremia: a rare Manifestation of COVID-19. Cureus 14 (4), e24090. 10.7759/cureus.24090 35573499 PMC9106551

[B105] Pawlik T. M. Olino K. Gleisner A. L. Torbenson M. Schulick R. Choti M. A. (2007). Preoperative chemotherapy for colorectal liver metastases: impact on hepatic histology and postoperative outcome. J. Gastrointest. Surg. Official J. Soc. For Surg. Alimentary Tract 11 (7), 860–868. 10.1007/s11605-007-0149-4 17492335

[B106] Pitot H. C. Goldberg R. M. Reid J. M. Sloan J. A. Skaff P. A. Erlichman C. (2000). Phase I dose-finding and pharmacokinetic trial of irinotecan hydrochloride (CPT-11) using a once-every-three-week dosing schedule for patients with advanced solid tumor malignancy. Clin. Cancer Res. Official J. Am. Assoc. For Cancer Res. 6 (6), 2236–2244. 10873073

[B107] Pommier Y. (2006). Topoisomerase I inhibitors: camptothecins and beyond. Nat. Rev. Cancer 6 (10), 789–802. 10.1038/nrc1977 16990856

[B108] Robinson S. M. Wilson C. H. Burt A. D. Manas D. M. White S. A. (2012). Chemotherapy-associated liver injury in patients with colorectal liver metastases: a systematic review and meta-analysis. Ann. Surg. Oncol. 19 (13), 4287–4299. 10.1245/s10434-012-2438-8 22766981 PMC3505531

[B109] Roshandel G. Ghasemi-Kebria F. Malekzadeh R. (2024). Colorectal cancer: epidemiology, risk factors, and prevention. Cancers 16 (8), 1530. 10.3390/cancers16081530 38672612 PMC11049480

[B110] Rothman K. J. Lanes S. Sacks S. T. (2004). The reporting odds ratio and its advantages over the proportional reporting ratio. Pharmacoepidemiol. Drug Saf. 13 (8), 519–523. 10.1002/pds.1001 15317031

[B111] Rougier P. Bugat R. Douillard J. Y. Culine S. Suc E. Brunet P. (1997). Phase II study of irinotecan in the treatment of advanced colorectal cancer in chemotherapy-naive patients and patients pretreated with fluorouracil-based chemotherapy. J. Clin. Oncol. Official J. Am. Soc. Clin. Oncol. 15 (1), 251–260. 10.1200/JCO.1997.15.1.251 8996150

[B112] Rougier P. Van Cutsem E. Bajetta E. Niederle N. Possinger K. Labianca R. (1998). Randomised trial of irinotecan versus fluorouracil by continuous infusion after fluorouracil failure in patients with metastatic colorectal cancer. Lancet London, Engl. 352 (9138), 1407–1412. 10.1016/S0140-6736(98)03085-2 9807986

[B113] Rowinsky E. K. Grochow L. B. Ettinger D. S. Sartorius S. E. Lubejko B. G. Chen T. L. (1994). Phase I and pharmacological study of the novel topoisomerase I inhibitor 7-ethyl-10-[4-(1-piperidino)-1-piperidino]carbonyloxycamptothecin (CPT-11) administered as a ninety-minute infusion every 3 weeks. Cancer Res. 54 (2), 427–436. 8275479

[B114] Sakaeda T. Tamon A. Kadoyama K. Okuno Y. (2013). Data mining of the public version of the FDA adverse event reporting system. Int. J. Med. Sci. 10 (7), 796–803. 10.7150/ijms.6048 23794943 PMC3689877

[B115] Sakakibara R. (2023). Gastrointestinal dysfunction in multiple Sclerosis and related conditions. Seminars Neurology 43 (4), 598–608. 10.1055/s-0043-1771462 37703888

[B116] Sasaki Y. Yoshida Y. Sudoh K. Hakusui H. Fujii H. Ohtsu T. (1995). Pharmacological correlation between total drug concentration and lactones of CPT-11 and SN-38 in patients treated with CPT-11. Jpn. J. Cancer Res. Gann 86 (1), 111–116. 10.1111/j.1349-7006.1995.tb02995.x 7737902 PMC5920577

[B117] Schumacher J. D. Guo G. L. (2015). Mechanistic review of drug-induced steatohepatitis. Toxicol. Appl. Pharmacol. 289 (1), 40–47. 10.1016/j.taap.2015.08.022 26344000 PMC4628855

[B118] Shimada Y. Yoshino M. Wakui A. Nakao I. Futatsuki K. Sakata Y. (1993). Phase II study of CPT-11, a new camptothecin derivative, in metastatic colorectal cancer. CPT-11 Gastrointestinal Cancer Study Group. CPT-11 Gastrointest. Cancer Study Group. J. Clin. Oncol. Official J. Am. Soc. Clin. Oncol. 11 (5), 909–913. 10.1200/JCO.1993.11.5.909 8487053

[B119] Shu Y. Chen J. Ding Y. Zhang Q. (2023). Adverse events with risankizumab in the real world: postmarketing pharmacovigilance assessment of the FDA adverse event reporting system. Front. Immunol. 14, 1169735. 10.3389/fimmu.2023.1169735 37256136 PMC10225532

[B120] Singh P. Chung H.-J. Lee I.-A. D'souza R. Kim H.-J. Hong S.-T. (2018). Elucidation of the anti-hyperammonemic mechanism of Lactobacillus amylovorus JBD401 by comparative genomic analysis. BMC Genomics 19 (1), 292. 10.1186/s12864-018-4672-3 29695242 PMC5918772

[B121] Smith H. S. Busracamwongs A. (2003). Management of hiccups in the palliative care population. Am. J. Hospice & Palliat. Care 20 (2), 149–154. 10.1177/104990910302000214 12693648

[B122] Su Y. Y. Chiang N. J. Chang J. S. Wang Y. W. Shen B. N. Li Y. J. (2023). The association between UGT1A1 polymorphisms and treatment toxicities of liposomal irinotecan. ESMO Open 8 (1), 100746. 10.1016/j.esmoop.2022.100746 36527823 PMC10024091

[B123] Sugimoto M. Takagi T. Suzuki R. Konno N. Asama H. Sato Y. (2021). Mirogabalin vs pregabalin for chemotherapy-induced peripheral neuropathy in pancreatic cancer patients. BMC Cancer 21 (1), 1319. 10.1186/s12885-021-09069-9 34886831 PMC8656082

[B124] Suzuki A. Kato M. (1996). Chemotherapeutic agent CPT-11 induces the new expression of the apoptosis initiator to the cytoplasm. Exp. Cell Res. 227 (1), 154–159. 10.1006/excr.1996.0260 8806462

[B125] Swerdlow A. J. Higgins C. D. Smith P. Cunningham D. Hancock B. W. Horwich A. (2011). Second cancer risk after chemotherapy for Hodgkin's lymphoma: a collaborative British cohort study. J. Clin. Oncol. Official J. Am. Soc. Clin. Oncol. 29 (31), 4096–4104. 10.1200/JCO.2011.34.8268 21969511

[B126] Szarfman A. Machado S. G. O'neill R. T. (2002). Use of screening algorithms and computer systems to efficiently signal higher-than-expected combinations of drugs and events in the US FDA's spontaneous reports database. Drug Saf. 25 (6), 381–392. 10.2165/00002018-200225060-00001 12071774

[B127] Szebeni J. Simberg D. González-Fernández Á. Barenholz Y. Dobrovolskaia M. A. (2018). Roadmap and strategy for overcoming infusion reactions to nanomedicines. Nat. Nanotechnol. 13 (12), 1100–1108. 10.1038/s41565-018-0273-1 30348955 PMC6320688

[B128] Takiguchi Y. Watanabe R. Nagao K. Kuriyama T. (2002). Hiccups as an adverse reaction to cancer chemotherapy. J. Natl. Cancer Inst. 94 (10), 772. 10.1093/jnci/94.10.772 12011230

[B129] Tang L. Li X. Wan L. Xiao Y. Zeng X. Ding H. (2019). Herbal Medicines for irinotecan-induced diarrhea. Front. Pharmacol. 10, 182. 10.3389/fphar.2019.00182 30983992 PMC6450188

[B130] Tanneau R. S. Pennec Y. L. Morin J. F. Codet J. P. Bourbigot B. Garre M. (1993). Salt wastage, plasma volume contraction and hypokalemic paralysis in self-induced water intoxication. Nephron 64 (4), 570–575. 10.1159/000187402 8366983

[B131] Tsavaris N. Ziras N. Kosmas C. Giannakakis T. Gouveris P. Vadiaka M. (2003). Two different schedules of irinotecan (CPT-11) in patients with advanced colorectal carcinoma relapsing after a 5-fluorouracil and leucovorin combination. A randomized study. Cancer Chemother. Pharmacol. 52 (6), 514–519. 10.1007/s00280-003-0659-z 14504920

[B132] Tsuboya A. Fujita K.-I. Kubota Y. Ishida H. Taki-Takemoto I. Kamei D. (2019). Coadministration of cytotoxic chemotherapeutic agents with irinotecan is a risk factor for irinotecan-induced cholinergic syndrome in Japanese patients with cancer. Int. J. Clin. Oncol. 24 (2), 222–230. 10.1007/s10147-018-1347-7 30244364

[B133] Tsuji K. Yasui H. Onozawa Y. Boku N. Doyama H. Fukutomi A. (2012). Modified FOLFOX-6 therapy for heavily pretreated advanced gastric cancer refractory to fluorouracil, irinotecan, cisplatin and taxanes: a retrospective study. Jpn. J. Clin. Oncol. 42 (8), 686–690. 10.1093/jjco/hys084 22693245

[B134] Uchiyama K. Saito Y. Takekuma Y. Yuki S. Sugawara M. (2021). Alleviation of abdominal pain due to irinotecan-induced cholinergic syndrome using loperamide: a case report. Case Rep. Oncol. 14 (2), 806–811. 10.1159/000516403 34248544 PMC8255746

[B135] Van Hasselt J. G. C. Rahman R. Hansen J. Stern A. Shim J. V. Xiong Y. (2020). Transcriptomic profiling of human cardiac cells predicts protein kinase inhibitor-associated cardiotoxicity. Nat. Commun. 11 (1), 4809. 10.1038/s41467-020-18396-7 32968055 PMC7511315

[B136] Van Puijenbroek E. P. Bate A. Leufkens H. G. M. Lindquist M. Orre R. Egberts A. C. G. (2002). A comparison of measures of disproportionality for signal detection in spontaneous reporting systems for adverse drug reactions. Pharmacoepidemiol. Drug Saf. 11 (1), 3–10. 10.1002/pds.668 11998548

[B137] Varga Z. V. Erdelyi K. Paloczi J. Cinar R. Zsengeller Z. K. Jourdan T. (2018). Disruption of Renal Arginine metabolism promotes Kidney injury in Hepatorenal syndrome in mice. Hepatology 68 (4), 1519–1533. 10.1002/hep.29915 29631342 PMC6173643

[B138] Vauthey J.-N. Pawlik T. M. Ribero D. Wu T.-T. Zorzi D. Hoff P. M. (2006). Chemotherapy regimen predicts steatohepatitis and an increase in 90-day mortality after surgery for hepatic colorectal metastases. J. Clin. Oncol. Official J. Am. Soc. Clin. Oncol. 24 (13), 2065–2072. 10.1200/JCO.2005.05.3074 16648507

[B139] Voûte P. A. (2000). Second malignant tumours. Ann. Oncol. Official J. Eur. Soc. For Med. Oncol. 11 (Suppl. 3), 79–82. 11079123

[B140] Wang Q. Zhou Q. Du Z. Lu R. Jiang Y. Zhu H. (2024). Clinical safety of daridorexant in insomnia treatment: analysis of FDA adverse event reports. J. Affect. Disord. 362, 552–559. 10.1016/j.jad.2024.07.034 39019232

[B141] Wang-Gillam A. Li C.-P. Bodoky G. Dean A. Shan Y.-S. Jameson G. (2016). Nanoliposomal irinotecan with fluorouracil and folinic acid in metastatic pancreatic cancer after previous gemcitabine-based therapy (NAPOLI-1): a global, randomised, open-label, phase 3 trial. Lancet London, Engl. 387 (10018), 545–557. 10.1016/S0140-6736(15)00986-1 26615328

[B142] Watanabe J. Terazawa T. Yamane S. Kazama H. Uetake H. Yoshino T. (2023). Aflibercept with FOLFIRI in Japanese patients with metastatic colorectal cancer: results of a post-marketing surveillance. Int. J. Clin. Oncol. 28 (1), 130–138. 10.1007/s10147-022-02259-w 36307632 PMC9823052

[B143] Wei W. Bai Y.-T. Chang E. Liu J.-F. (2024). Post-marketing safety surveillance of fostamatinib: an observational, pharmacovigilance study leveraging FAERS database. Expert Opin. Drug Saf., 1–9. 10.1080/14740338.2024.2387315 39078338

[B144] Willeke F. Horisberger K. Kraus-Tiefenbacher U. Wenz F. Leitner A. Hochhaus A. (2007). A phase II study of capecitabine and irinotecan in combination with concurrent pelvic radiotherapy (CapIri-RT) as neoadjuvant treatment of locally advanced rectal cancer. Br. J. Cancer 96 (6), 912–917. 10.1038/sj.bjc.6603645 17325705 PMC2360100

[B145] Williams C. D. Stengel J. Asike M. I. Torres D. M. Shaw J. Contreras M. (2011). Prevalence of nonalcoholic fatty liver disease and nonalcoholic steatohepatitis among a largely middle-aged population utilizing ultrasound and liver biopsy: a prospective study. Gastroenterology 140 (1), 124–131. 10.1053/j.gastro.2010.09.038 20858492

[B146] Wiseman L. R. Markham A. (1996). Irinotecan. A review of its pharmacological properties and clinical efficacy in the management of advanced colorectal cancer. Drugs 52 (4), 606–623. 10.2165/00003495-199652040-00013 8891470

[B147] Wu D. Cederbaum A. I. (2013). Inhibition of autophagy promotes CYP2E1-dependent toxicity in HepG2 cells via elevated oxidative stress, mitochondria dysfunction and activation of p38 and JNK MAPK. Redox Biol. 1 (1), 552–565. 10.1016/j.redox.2013.10.008 24273738 PMC3836279

[B148] Xu F. Ren X. Chen Y. Li Q. Li R. Chen Y. (2018). Irinotecan-platinum combination therapy for previously untreated extensive-stage small cell lung cancer patients: a meta-analysis. BMC Cancer 18 (1), 808. 10.1186/s12885-018-4715-9 30097029 PMC6086076

[B149] Xu S. Lan H. Huang C. Ge X. Zhu J. (2024). Mechanisms and emerging strategies for irinotecan-induced diarrhea. Eur. J. Pharmacol. 974, 176614. 10.1016/j.ejphar.2024.176614 38677535

[B150] Yahagi M. Tsuruta M. Hasegawa H. Okabayashi K. Kitagawa Y. (2017). Non-alcoholic fatty liver disease fibrosis score predicts hematological toxicity of chemotherapy including irinotecan for colorectal cancer. Mol. Clin. Oncol. 6 (4), 529–533. 10.3892/mco.2017.1177 28413661 PMC5374966

[B151] Yang X. Hu Z. Chan S. Y. Chan E. Goh B. C. Duan W. (2005). Novel agents that potentially inhibit irinotecan-induced diarrhea. Curr. Med. Chem. 12 (11), 1343–1358. 10.2174/0929867054020972 15975002

[B152] Yoo C. Kim K.-P. Jeong J. H. Kim I. Kang M. J. Cheon J. (2021). Liposomal irinotecan plus fluorouracil and leucovorin versus fluorouracil and leucovorin for metastatic biliary tract cancer after progression on gemcitabine plus cisplatin (NIFTY): a multicentre, open-label, randomised, phase 2b study. Lancet. Oncol. 22 (11), 1560–1572. 10.1016/S1470-2045(21)00486-1 34656226

[B153] Yoshino T. Boku N. Onozawa Y. Hironaka S. Fukutomi A. Yamaguchi Y. (2007). Efficacy and safety of an irinotecan plus bolus 5-fluorouracil and L-leucovorin regimen for metastatic colorectal cancer in Japanese patients: experience in a single institution in Japan. Jpn. J. Clin. Oncol. 37 (9), 686–691. 10.1093/jjco/hym091 17720736

[B154] Zhai C. Cai Y. Lou F. Liu Z. Xie J. Zhou X. (2018). Multiple primary malignant tumors - a clinical analysis of 15,321 patients with malignancies at a single center in China. J. Cancer 9 (16), 2795–2801. 10.7150/jca.25482 30123347 PMC6096360

[B155] Zhen D. B. Mcdevitt R. L. Zalupski M. M. Sahai V. (2019). Irinotecan-associated dysarthria: a single institution case series with management implications in patients with gastrointestinal malignancies. J. Oncol. Pharm. Pract. Official Publ. Int. Soc. Oncol. Pharm. Pract. 25 (4), 980–986. 10.1177/1078155218763044 29562843

[B156] Zhu X. Huang Y. Ding J. Liu J. Cui C. Han G. (2024). Investigating the impact of SN-38 on Mouse brain metabolism based on Metabolomics. Drug Des. Dev. Ther. 18, 2435–2447. 10.2147/DDDT.S457698 PMC11195675 38915864

[B157] Zhu Z. Li Y. Zhu C. Dong Q. Zhang Y. Liu Z. (2025). Disproportionality analysis of interstitial lung disease associated with novel antineoplastic agents during breast cancer treatment: a pharmacovigilance study. EClinicalMedicine 82, 103160. 10.1016/j.eclinm.2025.103160 40166653 PMC11957809

[B158] Zou S. Ouyang M. Zhao Y. Cheng Q. Shi X. Sun M. (2024). A disproportionality analysis of adverse events caused by GnRHas from the FAERS and JADER databases. Front. Pharmacol. 15, 1392914. 10.3389/fphar.2024.1392914 39027335 PMC11254796

