# Supplementary figures and images for: Safety evaluation of irinotecan: a real-world disproportionality analysis using FAERS and JADER databases during the time period 2004-2024
Source: Front Pharmacol. 2025 Jun 9;16:1516449. doi: 10.3389/fphar.2025.1516449 (PMC12184384; doi:10.3389/fphar.2025.1516449)

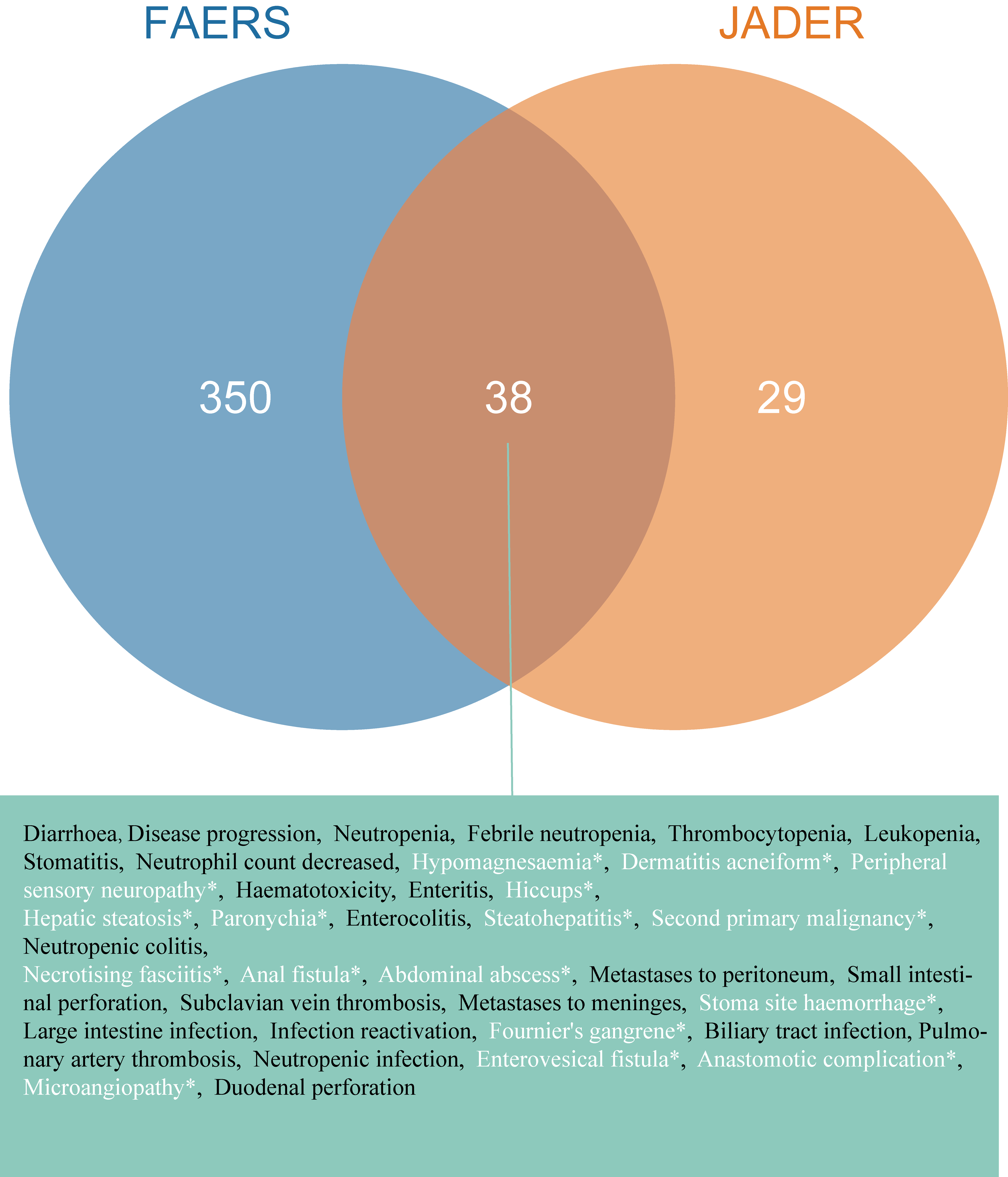

Supplement: Supplementary file 3 [file Image2.tif]

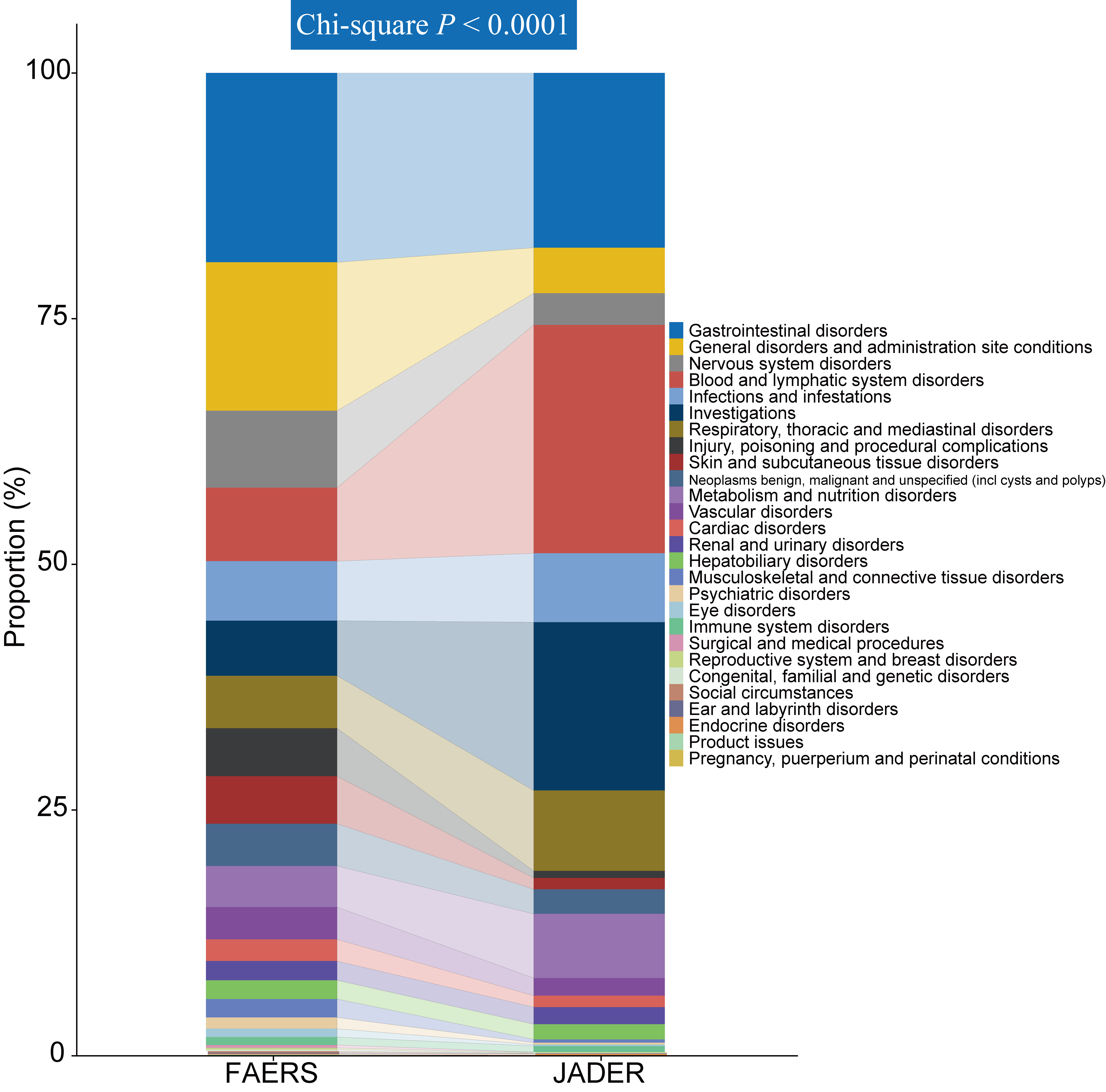

Supplement: Supplementary file 4 [file Image1.tif]
